# A prospective, randomized, single-blinded, crossover trial to investigate the effect of a wearable device in addition to a daily symptom diary for the remote early detection of SARS-CoV-2 infections (COVID-RED): a structured summary of a study protocol for a randomized controlled trial

**DOI:** 10.1186/s13063-021-05241-5

**Published:** 2021-06-22

**Authors:** Timo B. Brakenhoff, Billy Franks, Brianna Mae Goodale, Janneke van de Wijgert, Santiago Montes, Duco Veen, Eskild K. Fredslund, Theo Rispens, Lorenz Risch, Ariel V. Dowling, Amos A. Folarin, Patricia Bruijning, Richard Dobson, Tessa Heikamp, Paul Klaver, Maureen Cronin, Diederick E. Grobbee, On behalf of the COVID-RED Consortium, Spiros Denaxas, Johannes B. Reitsma, Christian Simon, Alison Kuchta, Pieter Stolk, George Downward, René van Lier, Jakob Kjellberg, Martin Risch, Kirsten Grossmann, David Conen, Stefanie Aeschbacher

**Affiliations:** 1 Julius Clinical, Zeist, the Netherlands; 2 Ava AG, Zurich, Switzerland; 3 Julius Center for Health Sciences and Primary Care, University Medical Center Utrecht, Utrecht, the Netherlands; 4 Roche Diagnostics Nederland BV, Almere, the Netherlands; 5 Julius Global Health, Julius Center for Health Sciences and Primary Care, University Medical Center Utrecht, Utrecht, the Netherlands; 6 Optentia Research Program, North-West University, Potchefstroom, South Africa; 7 Vive, Aarhus, Denmark; 8 Sanquin, Amsterdam, the Netherlands; 9 Labormedizinisches zentrum Dr. Risch, Vaduz, Liechtenstein; 10 Faculty of Medical Sciences, Private Universität im Fürstentum Liechtenstein, Triesen, Liechtenstein; 11 Center of Laboratory Medicine, University Institute of Clinical Chemistry, University of Bern, Bern, Switzerland; 12 Data Sciences Institute, Takeda Pharmaceuticals U.S.A. Inc., Cambridge, MA USA; 13 National Institute for Health Research Maudsley Biomedical Research Centre, King’s College London, London, UK; 14 Institute of Health Informatics, University College London, London, UK; 15 South London and Maudsley NHS Foundation Trust, London, UK; 16 https://www.covid-red.eu/

**Keywords:** COVID-19, Randomised controlled trial, protocol, wearable device, mobile application, early detection, SARS-CoV-2, prospective, physiological parameters, algorithm, machine learning, symptom diary

## Abstract

**Objectives:**

It is currently thought that most—but not all—individuals infected with SARS-CoV-2 develop symptoms, but that the infectious period starts on average two days before the first overt symptoms appear. It is estimated that pre- and asymptomatic individuals are responsible for more than half of all transmissions. By detecting infected individuals before they have overt symptoms, wearable devices could potentially and significantly reduce the proportion of transmissions by pre-symptomatic individuals.

Using laboratory-confirmed SARS-CoV-2 infections (detected via serology tests [to determine if there are antibodies against the SARS-CoV-2 in the blood] or SARS-CoV-2 infection tests such as polymerase chain reaction [PCR] or antigen tests) as the gold standard, we will determine the sensitivity, specificity, positive predictive value (PPV) and negative predictive value (NPV) for the following two algorithms to detect first time SARS-CoV-2 infection including early or asymptomatic infection:

- the algorithm using Ava bracelet data when coupled with self-reported Daily Symptom Diary data (Wearable + Symptom Data Algo; experimental condition)
- the algorithm using self-reported Daily Symptom Diary data alone (Symptom Only Algo; control condition)

In addition, we will determine which of the two algorithms has superior performance characteristics for detecting SARS-CoV-2 infection including early or asymptomatic infection as confirmed by SARS-CoV-2 virus testing.

**Trial design:**

The trial is a randomized, single-blinded, two-period, two-sequence crossover trial. All subjects will participate in an initial Learning Phase (varying from 2 weeks to 3 months depending on enrolment date), followed by two contiguous 3-month test phases, Period 1 and Period 2. Each subject will undergo the experimental condition (the Wearable + Symptom Data Algo) in one of these periods and the control condition (Symptom Only Algo) in the other period. The order will be randomly assigned, resulting in subjects being allocated 1:1 to either Sequence 1 (experimental condition first) or Sequence 2 (control condition first). Based on demographics, medical history and/or profession, each subject will be stratified at baseline into a high-risk and normal-risk group within each sequence.

**Participants:**

The trial will be conducted in the Netherlands. A target of 20,000 subjects will be enrolled. Based on demographics, medical history and/or profession, each subject will be stratified at baseline into a high-risk and normal-risk group within each sequence. This results in approximately 6,500 normal-risk individuals and 3,500 high-risk individuals per sequence. Subjects will be recruited from previously studied cohorts as well as via public campaigns and social media. All data for this study will be collected remotely through the Ava COVID-RED app, the Ava bracelet, surveys in the COVID-RED web portal, and self-sampling serology and PCR kits. During recruitment, subjects will be invited to visit the COVID-RED web portal (www.covid-red.eu). After successfully completing the enrolment questionnaire, meeting eligibility criteria and indicating interest in joining the study, subjects will receive the subject information sheet and informed consent form. Subjects can enrol in COVID-RED if they comply with the following inclusion and exclusion criteria.

Inclusion criteria:

- Resident of the Netherlands
- At least 18 years old
- Informed consent provided (electronic)
- Willing to adhere to the study procedures described in the protocol
- Must have a smartphone that runs at least Android 8.0 or iOS 13.0 operating systems and is active for the duration of the study (in the case of a change of mobile number, study team should be notified)
- Be able to read, understand and write Dutch

Exclusion criteria:

- Previous positive SARS-CoV-2 test result (confirmed either through PCR/antigen or antibody tests; self-reported)
- Previously received a vaccine developed specifically for COVID-19 or in possession of an appointment for vaccination in the near future (self-reported)
- Current suspected (e.g., waiting for test result) COVID-19 infection or symptoms of a COVID-19 infection (self-reported)
- Participating in any other COVID-19 clinical drug, vaccine, or medical device trial (self-reported)
- Electronic implanted device (such as a pacemaker; self-reported)
- Pregnant at time of informed consent (self-reported)
- Suffering from cholinergic urticaria (per the Ava bracelet’s User Manual; self-reported)
- Staff involved in the management or conduct of this study

**Intervention and comparator:**

All subjects will be instructed to complete the Daily Symptom Diary in the Ava COVID-RED app daily, wear their Ava bracelet each night and synchronise it with the app each day for the entire period of study participation. Provided with wearable sensor and/or self-reported symptom data within the last 24 hours, the Ava COVID-RED app’s underlying algorithms will provide subjects with a real-time indicator of their overall health and well-being. Subjects will see one of three messages, notifying them that: no seeming deviations in symptoms and/or physiological parameters have been detected; some changes in symptoms and/or physiological parameters have been detected and they should self-isolate; or alerting them that deviations in their symptoms and/or physiological parameters could be suggestive of a potential COVID-19 infection and to seek additional testing. We will assess intraperson performance of the algorithms in the experimental condition (Wearable + Symptom Data Algo) and control conditions (Symptom Only Algo).

**Main outcomes:**

The trial will evaluate the use and performance of the Ava COVID-RED app and Ava bracelet, which uses sensors to measure breathing rate, pulse rate, skin temperature, and heart rate variability for the purpose of early and asymptomatic detection and monitoring of SARS-CoV-2 in general and high-risk populations.

Using laboratory-confirmed SARS-CoV-2 infections (detected via serology tests, PCR tests and/or antigen tests) as the gold standard, we will determine the sensitivity, specificity, positive predictive value (PPV) and negative predictive value (NPV) for each of the following two algorithms to detect first-time SARS-CoV-2 infection including early or asymptomatic infection: the algorithm using Ava Bracelet data when coupled with the self-reported Daily Symptom Diary data, and the algorithm using self-reported Daily Symptom Diary data alone. In addition, we will determine which of the two algorithms has superior performance characteristics for detecting SARS-CoV-2 infection including early or asymptomatic infection as confirmed by SARS-CoV-2 virus testing. The protocol contains an additional seventeen secondary outcomes which address infection incidence rates, health resource utilization, symptoms reported by SARS-CoV-2 infected participants, and the rate of breakthrough and asymptomatic SARS-CoV-2 infections among individuals vaccinated against COVID-19.

PCR or antigen testing will occur when the subject receives a notification from the algorithm to seek additional testing. Subjects will be advised to get tested via the national testing programme, and report the testing result in the Ava COVID-RED app and a survey. If they cannot obtain a test via the national testing programme, they will receive a nasal swab self-sampling kit at home, and the sample will be tested by PCR in a trial-affiliated laboratory. In addition, all subjects will be asked to take a capillary blood sample at home at baseline (Month 0), and at the end of the Learning Phase (Month 3), Period 1 (Month 6) and Period 2 (Month 9). These samples will be used for SARS-CoV-2-specific antibody testing in a trial-affiliated laboratory, differentiating between antibodies resulting from a natural infection and antibodies resulting from COVID-19 vaccination (as vaccination will gradually be rolled out during the trial period). Baseline samples will only be analysed if the sample collected at the end of the Learning Phase is positive, and samples collected at the end of Period 1 will only be analysed if the sample collected at the end of Period 2 is positive. When subjects obtain a positive PCR/antigen or serology test result during the study, they will continue to be in the study but will be moved into a so-called “COVID-positive” mode in the Ava COVID-RED app. This means that they will no longer receive recommendations from the algorithms but can still contribute and track symptom and bracelet data.

The primary analysis of the main objective will be executed using data collected in Period 2 (Month 6 through 9). Within this period, serology tests (before and after Period 2) and PCR/antigen tests (taken based on recommendations by the algorithms) will be used to determine if a subject was infected with SARS-CoV-2 or not. Within this same time period, it will be determined if the algorithms gave any recommendations for testing. The agreement between these quantities will be used to evaluate the performance of the algorithms and how these compare between the study conditions.

**Randomisation:**

All eligible subjects will be randomized using a stratified block randomization approach with an allocation ratio of 1:1 to one of two sequences (experimental condition followed by control condition or control condition followed by experimental condition). Based on demographics, medical history and/or profession, each subject will be stratified at baseline into a high-risk and normal-risk group within each sequence, resulting in equal numbers of high-risk and normal-risk individuals between the sequences.

**Blinding (masking):**

In this study, subjects will be blinded as to study condition and randomization sequence. Relevant study staff and the device manufacturer will be aware of the assigned sequence. The subject will wear the Ava bracelet and complete the Daily Symptom Diary in the Ava COVID-RED app for the full duration of the study, and they will not know if the feedback they receive about their potential infection status will only be based on data they entered in the Daily Symptom Diary within the Ava COVID-RED app or based on both the data from the Daily Symptom Diary and the Ava bracelet.

**Numbers to be randomised (sample size):**

20,000 subjects will be recruited and randomized 1:1 to either Sequence 1 (experimental condition followed by control condition) or Sequence 2 (control condition followed by experimental condition), taking into account their risk level. This results in approximately 6,500 normal-risk and 3,500 high-risk individuals per sequence.

**Trial Status:**

Protocol version: 1.2, dated January 22^nd^, 2021

Start of recruitment: February 22^nd^, 2021

End of recruitment (estimated): April 2021

End of follow-up (estimated): December 2021

**Trial registration:**

The trial has been registered at the Netherlands Trial Register on the 18^th^ of February, 2021 with number NL9320 (https://www.trialregister.nl/trial/9320)

**Full protocol:**

The full protocol is attached as an additional file, accessible from the Trials website (Additional file 1). In the interest in expediting dissemination of this material, the familiar formatting has been eliminated; this Letter serves as a summary of the key elements of the full protocol.

**Supplementary Information:**

The online version contains supplementary material available at 10.1186/s13063-021-05241-5.

## Supplementary Information

**Additional file 1.** Full study protocol.

## Data Availability

The final trial dataset will be available for consortium members, and specific parts of the pseudonymized data will be made available according to the FAIR principles for external researchers that need the research data to address the public health emergency. However, no special access rights for third parties shall be granted to personal data or data of commercial sensitivity.

