# Supplementary material for: A prospective, randomized, single-blinded, crossover trial to investigate the effect of a wearable device in addition to a daily symptom diary for the remote early detection of SARS-CoV-2 infections (COVID-RED): a structured summary of a study protocol for a randomized controlled trial
Source: Trials. 2021 Jun 22;22:412. doi: 10.1186/s13063-021-05241-5 (PMC8218271; doi:10.1186/s13063-021-05241-5)
Supplement: Supplementary file 1 — Additional file 1. Full study protocol. [file 13063_2021_5241_MOESM1_ESM.docx]

**PROTOCOL TITLE** COVID-19 infections – Remote Early Detection. A prospective, randomized, single-blinded, crossover trial to investigate the added benefit of a wearable on top of a daily symptom diary for the (early) detection of SARS-CoV-2 infections

| **Short title** | **COVID-RED** |
| --- | --- |
| **EudraCT number** | **Not Applicable** |
| **Version** | **1.2** |
| **Date** | **22Jan2021** |
| **Principal investigator/project leader** | **Prof. Diederick Grobbee, MD, PhD, FESC** |
| **Additional key study team members** | **Maureen Cronin, MD, PhD**  **Brianna Goodale, PhD**  **Lorenz Risch, MD, PhD**  **Martin Risch, PhD**  **Santiago Montes, MBA**  **René van Lier, MD, PhD**  **Theo Rispens, PhD** |
|  | **Ariel Dowling, PhD**  **Prof. Janneke van de Wijgert, MD, PhD, MPH**  **Hans Reitsma, MD, PhD**  **Patricia Bruijning, MD, PhD**  **Prof. Richard Dobson, PhD**  **Amos Folarin, BSc, MRes, PhD**  **Eskild Fredslund, PhD**  **Prof. Jakob Kjellberg, cand. Sceient, MSc**  **Timo Brakenhoff, PhD**  **Duco Veen, PhD**  **Billy Franks, PhD**  **Christian Simon, PhD**  **Alison Kuchta, MD, PhD** |
| **Sponsor (in Dutch: verrichter/opdrachtgever)** | **Universitair Medisch Centrum Utrecht (UMCU), Julius Center**  **Postbus 85500**  **3508 GA Utrecht** |
|  | **Dr. Pieter Stolk**  **p.stolk@umcutrecht.nl** |
| **Subsidising party** | **H2020** |
| **Independent expert(s)** | **Tamara Platteel, MD, PhD, MSc** |
| **Central lab** | **Sanquin**  **Plesmanlaan 125**  **1066 CX Amsterdam**  **The Netherlands** |

**PROTOCOL SIGNATURE SHEET**

| **Name** | **Signature** | **Date** |
| --- | --- | --- |
| **Sponsor legal representative/Head of department:**  Carl Moons |  |  |
| **Principal Investigator:**  Prof. Diederick Grobbee, MD, PhD, FESC |  |  |

**TABLE OF CONTENTS**

1. INTRODUCTION AND RATIONALE 11

1.1 Artificial intelligence (AI) technologies to support monitoring systems 12

1.2 Mobile phone applications (apps) 12

1.3 Wearable sensor technology 13

1.4 COVID-RED project summary 13

2. OBJECTIVES 16

2.1 Primary Objectives: 16

2.2 Secondary Objective(s): 16

2.3 Exploratory Objective(s): 17

3. STUDY DESIGN 18

3.1 Design 18

3.2 Data collection 18

3.3 Implementation and development of algorithms 19

3.4 Algorithm indicators 20

3.5 SARS-CoV-2 infection testing 21

3.6 Serology testing for SARS-CoV-2 22

3.7 COVID-19 Vaccines 22

3.8 Additional outcome measures 22

4. STUDY POPULATION 26

4.1 Population (base) 26

4.2 Inclusion criteria 26

4.3 Exclusion criteria 26

4.4 Sample size calculation 27

4.4.1 Ever-infected analysis 28

4.4.2 Early detection analysis 29

5. TREATMENT OF SUBJECTS 31

5.1 Investigational product/treatment 31

5.2 Use of co-intervention (if applicable) 31

5.3 Escape medication (if applicable) 31

6. INVESTIGATIONAL PRODUCT 32

6.1 Name and description of investigational product(s) 32

6.2 Summary of findings from non-clinical studies 34

6.3 Summary of findings from clinical studies 34

6.4 Summary of known and potential risks and benefits 34

6.5 Description and justification of route of administration and dosage 35

6.6 Dosages, dosage modifications and method of administration 35

6.7 Preparation and labelling of Investigational Device 35

6.8 Device accountability 35

7. NON-INVESTIGATIONAL PRODUCT 36

8. METHODS 37

8.1 Study parameters/endpoints 37

8.1.1 Main study parameter/endpoint 37

8.1.2 Secondary study parameters/endpoints (if applicable) 37

8.1.3 Other study parameters 40

8.2 Randomisation, blinding and treatment allocation 42

8.3 Recruitment planning 42

8.4 Study procedures 43

8.4.1 (E)mail invitation to participate in COVID-RED 43

8.4.2 Registration and verification of inclusion/exclusion criteria 43

8.4.3 Informed consent by subject 43

8.4.4 Baseline survey 43

8.4.5 Assignment of Unique Identification code (UID) and randomization to sequence 44

8.4.6 Receive starter kit 44

8.4.7 Installation of the Ava COVID-RED app, log in and pairing of Ava bracelet 44

8.4.8 Collection & testing of plasma/blood sample 44

8.4.9 Daily logging of symptoms and confounders in Ava COVID-RED app 45

8.4.10 Nightly wearing & routine syncing of the Ava bracelet 45

8.4.11 Receive algorithm-based health indicator 45

8.4.12 Testing for presence of SARS-CoV-2 virus 46

8.4.13 Logging any SARS-CoV-2 test results and vaccination in Ava COVID-RED app 46

8.4.14 Periodic completion of survey 47

8.4.15 Regular completion of hospital episodes and adverse device events (ADEs) 47

8.4.16 Interview with subject for Healthcare Resource Utilization (HRU) and Adverse event (AE) reporting 47

8.4.17 Return of Ava bracelet 48

8.5 Withdrawal of individual subjects 50

8.5.1 Specific criteria for withdrawal 50

8.6 Replacement of individual subjects after withdrawal 50

8.7 Follow-up of subjects withdrawn from treatment 50

8.8 Premature termination of the study 50

9. SAFETY REPORTING 51

9.1 Temporary halt for reasons of subject safety 51

9.2 ADEs, SADEs and USADEs 51

9.2.1 Adverse device effects (ADEs) 51

9.2.2 Serious adverse device effects (SADEs) 51

9.2.3 Unanticipated serious adverse device effects (USADEs) 53

9.3 Annual safety report 53

9.4 Follow-up of adverse device effects 53

9.5 Data Safety Monitoring Board (DSMB) / Safety Committee 53

10. STATISTICAL ANALYSIS 54

10.1 Baseline Summary 56

10.2 Primary Analysis of Primary Endpoint(s) 56

10.3 Secondary Analysis of Primary Endpoint(s) 57

10.4 Synthesis of evidence 58

10.4.1 Bootstrap approach 59

10.4.2 Weighting of evidence 59

10.5 Analysis of Secondary Endpoint(s) 60

10.6 Analysis of Safety 62

10.7 Interim analysis 62

11. ETHICAL CONSIDERATIONS 63

11.1 Regulation statement 63

11.2 Recruitment and consent 63

11.3 Objection by minors or incapacitated subjects (if applicable) 64

11.4 Benefits and risks assessment, group relatedness 64

11.5 Compensation for injury 64

11.6 Incentives (if applicable) 64

12. ADMINISTRATIVE ASPECTS, MONITORING AND PUBLICATION 65

12.1 Handling and storage of data and documents 65

12.2 Monitoring and Quality Assurance 66

12.3 Amendments 67

12.4 Annual progress report 67

12.5 Temporary halt and (premature) end of study report 67

12.6 Public disclosure and publication policy 67

13. STRUCTURED RISK ANALYSIS 68

13.1 Potential issues of concern 68

13.2 Synthesis 70

14. REFERENCES 71

15. VERSION HISTORY 75

16. APPENDIX 1 76

17. APPENDIX 2 78

**LIST OF ABBREVIATIONS AND RELEVANT DEFINITIONS**

| **AE**  **ADE** | **Adverse Event**  **Adverse Device Effect** |
| --- | --- |
| **AI** | **Artificial Intelligence** |
| **BLE** | **Bluetooth Low Energy** |
| **DSMB** | **Data Safety Monitoring Board** |
| **EMA** | **European Medicines Agency** |
| **EU** | **European Union** |
| **EudraCT** | **European drug regulatory affairs Clinical Trials** |
| **GDPR** | **General Data Protection Regulation; in Dutch: Algemene Verordening Gegevensbescherming (AVG)** |
| **GGD** | **Municipal Health service; in Dutch: Gemeentelijke Gezondheidsdienst** |
| **GP** | **General Practitioner** |
| **HIV** | **Human Immunodeficiency Viruses** |
| **HRU** | **Health Resource Utilization** |
| **ICF** | **Informed Consent Form** |
| **ICU** | **Intensive Care Unit** |
| **IMDD** | **Investigational Medical Device Dossier** |
| **JC** | **Julius Clinical** |
| **NPV** | **Negative Predictive Value** |
| **PCR** | **Polymerase Chain Reaction** |
| **PPV** | **Positive Predictive Value** |
| **QMS** | **Quality Management System** |
| **RIVM** | **National Institute for Public Health and the Environment; in Dutch: Rijksinstituut voor Volksgezondheid en Milieu** |
| **(S)AE**  **SADE** | **(Serious) Adverse Event**  **Serious Adverse Device Effect** |
| **Sponsor** | **The sponsor is the party that commissions the organisation or performance of the research, for example a pharmaceutical**  **company, academic hospital, scientific organisation or investigator. A party that provides funding for a study but does not commission it is not regarded as the sponsor, but referred to as a subsidising party.** |
| **SUSAR** | **Suspected Unexpected Serious Adverse Reaction** |
| **UID** | **Unique Identification Code** |
| **WHO** | **World Health Organization** |
| **WHO-DD** | **World Health Organization Drug Dictionary** |

**SUMMARY**

**Rationale:** The World Health Organization (WHO) has declared the current coronavirus disease (COVID-19) outbreak, caused by the SARS-CoV-2 virus, to be a pandemic and, therefore, a Public Health Emergency of International Concern. The COVID-19 outbreak has a huge impact on health care, but also on economic and social costs. Track-and-trace programs are important measures to control the virus, but they have their limitations such as delays in the test results (e.g., it takes time to develop symptoms after infection, to access a test, receive the test result, and for close contacts to be traced). Early traceability of the virus may help in the track-and-trace programs to control the virus.

It is currently thought that most – but not all – infected individuals develop symptoms, but that the infectious period starts on average two days before the first overt symptoms appear. It is estimated that pre- and asymptomatic individuals are responsible for up to half of all transmissions. By detecting infected individuals before they have overt symptoms, the proportion of transmissions by pre-symptomatic individuals could potentially be significantly reduced.

**Primary Objective**:

Using laboratory-confirmed SARS-CoV-2 infections (detected via serology, PCR and/or antigen tests) as the gold standard, we will determine the sensitivity, specificity, positive predictive value (PPV) and negative predictive value (NPV) for each of the following two algorithms to detect *first time* SARS-CoV-2 infection including early or asymptomatic infection: the algorithm using Ava bracelet data when coupled with self-reported Daily Symptom Diary data, and the algorithm using self-reported Daily Symptom Diary data alone. In addition, we will determine which of the two algorithms has superior performance characteristics for detecting SARS-CoV-2 infection including early or asymptomatic infection as confirmed by SARS-CoV-2 virus testing.

**Study design:** Randomized, single-blinded, two-period, two-sequence crossover trial. All subjects will participate in an initial Learning Phase (2 weeks to 3 months), followed by a 3-month Period 1 and a 3-month Period 2. Each subject will undergo the experimental condition (=algorithm uses data from app and bracelet) in one of these periods and the control condition (=algorithm uses data from the app only) in the other period, but the order will be randomly assigned, resulting in Sequence 1 (experimental condition first) and Sequence 2 (control condition first).

**Study population:**

A target of 20,000 subjects will be enrolled in this study. Subjects will be recruited from previously studied cohorts as well as via public campaigns. They will be invited to visit the COVID-RED web portal. When they have successfully completed the survey questions in the COVID-RED web portal, are considered eligible and have indicated interest in joining the study, then they will receive the subject information sheet and consent form. Subjects can be enrolled when they comply with the following inclusion and exclusion criteria:

Key Inclusion criteria:

- Resident of the Netherlands
- At least 18 years old
- Must have a smartphone that runs at least Android 8.0 or iOS 13.0 operating systems and is active for the duration of the study (in the case of a change of mobile number, study team should be notified)
- Be able to read, understand and write Dutch

Key Exclusion criteria

- Previous positive SARS-CoV-2 test result (confirmed either through PCR/antigen or antibody tests) (self-reported)
- Previously received a vaccine developed specifically for COVID-19 or in possession of an appointment for vaccination in the near future
- Current suspected (e.g., waiting for test result) coronavirus infection or symptoms of a coronavirus infection (self-reported)
- Electronic implanted device (such as a pacemaker)
- Suffering from cholinergic urticaria

**Intervention**: All subjects will be instructed to complete the Daily Symptom Diary in the Ava COVID-RED app, wear their Ava bracelet each night and synchronise it with the app each day, during the entire period of study participation. The experimental condition (=algorithm uses app and bracelet data) will be compared to the control condition (=algorithm uses app data only).

**Main study parameters/endpoints:**

The primary endpoint for this study for each subject is the daily indication of potential SARS-CoV-2 infection as provided by the algorithm of the Ava COVID-RED app with or without using data from the Ava bracelet. This daily endpoint will be compared with actual SARS-CoV-2 test results (PCR/antigen and/or serology) collected before, during and at the end of study participation. For the primary comparison, this daily endpoint will be summarized over each trial period per subject to determine (1) whether a subject was ever judged to have had a high risk for a potential SARS-Cov-2 infection, and (2) whether a subject was ever confirmed to have had a SARS-CoV-2 infection by PCR/antigen and/or serology testing. For this comparison, parameters such as sensitivity, specificity, positive predictive value, and negative predictive value will be calculated.

**Nature and extent of the burden and risks associated with participation, benefit and group relatedness:** Subjects wearing the Ava bracelet may experience skin irritation or sensitization due to rubbing and friction. Subjects are instructed to only wear the device at night to allow the skin to dry and breath during the day. They will be instructed to discontinue wearing the Ava bracelet and contact the study team in case they experience any signs of allergic reaction, feel soreness, tingling, numbness, burning or stiffness in their hands or wrists while or after wearing the Ava bracelet. Subjects may feel uncomfortable to answer health questions in the Ava COVID-RED app but they have the choice of not responding to the questions in the app. Subjects will be asked to donate fingerprick blood for SARS-CoV-2 antibody testing at 4 different timepoints, which may cause minor discomfort. This study will use the existing testing infrastructure in the Netherlands provided by the Municipal Health services (GGD) for SARS-CoV-2 infection, and only when this is not possible PCR testing in the central study laboratory will be arranged. Recruitment and follow-up will be completely remote and take place via post, email, phone and electronic web portals. In this way, risk of SARS-CoV-2 infection is minimized as much as possible for those wanting to participate in the trial and for the staff conducting the trial.

Another risk for the subject is the potential breach of data security. The study team will implement security measures to prevent loss of data or unauthorised access to the data and we will follow the General Data Protection Regulation. Data will be pseudo-anonymized within the platforms where data analysis will be performed. Data transfers will use a trial-specific identifier which is not linked to any external participant identifiers.

Overall, the burden for the subjects is assessed as small and is justified given the importance of assessing a potential method in early detection of COVID-19. The expected benefit is large as the algorithms trained on the obtained data recordings from the Ava bracelet are expected to recognize COVID-19 earlier than the presentation of clinical symptoms. The latter would allow for earlier isolation and stratification as well as monitoring of SARS-CoV-2 infected persons preventing further spread and, if applicable, allowing for appropriate healthcare.

# INTRODUCTION AND RATIONALE

The World Health Organization (WHO) has declared the current coronavirus disease (COVID-19) outbreak, caused by the SARS-CoV-2 virus, to be a pandemic and, therefore, a Public Health Emergency of International Concern. In the first half of 2020, many governments worldwide implemented general measures (such as physical distancing, hygiene measures, and face masks) and lockdowns (closures of schools, businesses, public transport, and cancellation of events) to contain their epidemics, usually with success but at great economic and social costs. Once hospitals were no longer overcrowded, and viral transmission reduced, many of these governments implemented track-and-trace programs in an effort to continue controlling the virus but limiting the collateral damage. These track-and-trace programs generally start with testing of persons with COVID-19 symptoms (while asking them to self-quarantine until the test result is available), and if the test result is positive, by tracing their close contacts (who are also asked to self-quarantine, and in some cases, to be tested). Unfortunately, these systems are often fraught with incompleteness (e.g., not everyone gets tested or adheres to quarantine rules; not all close contacts can be traced) and delays (e.g., it takes time to develop symptoms after infection, to access a test, receive the test result, and for close contacts to be traced). Mathematical studies have shown that epidemic waves can only be stopped by testing-and-tracing if delays in the system are minimized (1). The main purpose of the COVID-RED project is to determine if daily vital signs monitoring via a wearable device over-and-above daily symptom-reporting via a mobile phone app, can detect COVID-19 earlier than symptom-reporting alone. This may help to reduce delays in public health track-and-trace systems.

It is currently thought that most – but not all - infected individuals develop symptoms, but that the infectious period starts on average two days before the first overt symptoms appear (2). It is estimated that pre- and asymptomatic individuals are responsible for up to half of all transmissions (3,4). Studies on COVID-19 reported that fever (87.9% of cases), dry cough (67.7% of cases), and shortness of breath (18.6% of cases) are the most frequent presenting symptoms (5–8). Close to half (44%) of infected Chinese patients reported to treatment centres with fever as their first presenting symptom (9). While not specific to COVID-19, body temperature and pulse rate increase during fever (10,11) and shortness of breath can be measured by increased breathing rate. A recent paper examining the validity of wrist temperatures compared to forehead and tympanic temperatures among Chinese COVID-19 patients found less overall variability in wrist temperatures (10). Increases in temperature, pulse rate, and breathing rate could therefore be early signs of a SARS-CoV-2 infection, especially if exposure to SARS-CoV-2 is known to have occurred (10). By detecting infected individuals before they have overt symptoms, the proportion of transmissions by presymptomatic individuals could potentially be significantly reduced. As of December 2020, the first of likely several vaccines developed specifically for COVID-19 have been approved by regulatory authorities. Like many other countries, the government of the Netherlands is aiming to vaccinate the large majority of the Dutch population by the end of 2021. Given the differences in effectiveness between manufactured vaccines, the uncertainties surrounding the duration of protection afforded by these vaccines, as well as the difficulty in vaccinating a large majority of the population in a timely manner, the (early) detection of COVID-19 remains a crucial component to contain the pandemic. While vaccinated individuals will not be eligible for this study, subject who receive a vaccine during follow-up will remain in the study and will contribute valuable data for understanding vaccines’ long-term efficacy.

## Artificial intelligence (AI) technologies to support monitoring systems

Traditionally, continuous monitoring of vital signs is used to facilitate early detection of patient deterioration, thereby ensuring timely management of disease (12–14). Continuous monitoring has primarily been used in intensive care units (ICU) but has recently made its way into general wards and even into patients’ own homes (12,15,16). Concurrently, an increasing interest for integrating artificial intelligence (AI) technologies to support continuous monitoring systems has evolved (14,17–21). AI is a rapidly growing field and AI technologies are increasingly being used or tested for different purposes in the health care sector. AI-supported health care technologies have proved to be comparable to human approaches by clinicians and some studies even point to increased accuracy of diagnosis and risk prediction (22,23). In this project, we will harness recent advances in AI and wearable sensor technology to detect potential COVID-19 infection prior to symptom development using a mobile phone application and wearable device.

## Mobile phone applications (apps)

Mobile health apps are increasingly gaining popularity: of the 3,195,204 active mobile apps available in the iTunes app store and the 3,612,250 active apps in the Google Play store in 2018, 95,851 and 105,912, respectively, were categorized as Health and Fitness (24). Mobile apps have been shown to successfully facilitate the self-management of chronic disease (25) and have been used for triage in the COVID-19 epidemic (26–28). In addition, mobile health apps have also been shown to be efficient tools of health communication (29–33).

## Wearable sensor technology

In recent years, wearables, such as smartwatches and fitness wristbands, have grown in popularity and have gained traction within the healthcare sector for real-time patient monitoring (34). Wearable devices have evolved gradually in the form of accessories, integrated clothing, body attachments and body inserts. The advanced sensors and increased connectedness of these devices will allow individuals to monitor and manage their own health with ever-greater accuracy . The investigation of the potential of available wearable technologies in the detection and monitoring of COVID-19 patients by the scientific community has recently been emphasized (35).

## COVID-RED project summary

In this project, we will evaluate the use and performance of the Ava bracelet, a device that uses three sensors to measure the wearers’ breathing rate, pulse rate, skin temperature, perfusion and heart rate variability while they sleep. Initially developed to detect menstrual cycle changes among ovulating women for which it is CE-marked (36–38), the bracelet’s underlying technology and hardware are gender agnostic; we aim to test whether this wearable device could be repurposed and its algorithms customized to detect pre-symptomatic COVID-19 infection in general and high-risk populations based on deviations in measured physiological parameters. The Ava bracelet integrates with a complementary mobile application (Ava-COVID) which also allows users to log any symptoms they may be experiencing along with potential confounding factors in a Daily Symptom Diary. Within the app, machine learning algorithms based solely on data from the Daily Symptom Diary (control condition) or on data from both the Daily Symptom Diary and the Ava bracelet (experimental condition) will provide real-time feedback to the subjects about potential infection status (36–38). We hypothesize that the algorithm based on the Daily Symptom Diary and bracelet in combination will perform better at detecting SARS-CoV-2 infection in general and, in the case of symptomatic infection, earlier than the algorithm based solely on the Daily Symptom Diary.

In this randomized, single-blind, 2-period crossover study we will follow 20,000 subjects in the Netherlands who will each log symptoms and wear the Ava bracelet up to 9 months. All subjects will participate in an initial Learning Phase, followed by a 3-month Period 1 and a 3-month Period 2. Each subject will undergo the control condition in one of these periods and the experimental condition in the other period, but the order will be randomly assigned. 10,000 subjects will be assigned to each period sequence stratified by risk-level, with approximately 6,500 from a general (normal-risk) population and 3,500 from a high-risk population (see Table 1).

During the study, the algorithms in both the control and experimental conditions will provide real-time prediction to subjects whether they likely have an active SARS-CoV-2 infection. Individuals with a suspected SARS-CoV-2 infection based on these algorithms will be encouraged to get tested using the existing testing infrastructure in the Netherlands provided by the Municipal Health services (GGD). At the moment of writing, the GGD offers free SARS-CoV-2 polymerase chain reaction (PCR) and antigen testing services. While in this protocol tests to confirm current SARS-CoV-2 infection are described as PCR and/or antigen tests, results from other validated testing methods used by the GGD will also be documented and processed in the same way, should the GGD choose to expand the type of tests being used to detect SARS-CoV-2 infection in Dutch residents. Subjects who are thought to have an SARS-CoV-2 infection according to the algorithm of the control or experimental condition, but are rejected for testing by the GGD, will be advised to use self-sampling kits provided by the study team which they can send to the central laboratory for testing. A reason for such rejection by the GGD may include not (yet) having overt symptoms of a respiratory tract infection plus not having any evidence (such as a GGD or CoronaMelder app notification) of having been in close contact with someone who tested SARS-CoV-2-positive. In addition, fingerstick capillary blood samples will be collected from each COVID-RED subject at baseline, after the Learning Phase and after the completion of each period. Samples collected after the Learning Phase and the completion of Period 2 will be tested for SARS-CoV-2 antibodies to determine whether the subject had a previous SARS-CoV-2 infection prior to the study or during the test periods (which was not detected through real-time SARS-CoV-2 infection testing). Baseline samples of subjects will only be analysed if the sample collected at the end of the Learning Phase is positive for SARS-CoV-2 antibodies. Samples of subjects collected at the end of Period 1 will only be analysed if the sample collected at the end of Period 2 is positive for SARS-CoV-2 antibodies.

This design and infection testing strategy will enable a proper evaluation of the algorithms in the setting of asymptomatic cases, pre-symptomatic cases, or when individuals lack respiratory symptoms. The sensitivity and specificity of the algorithms used in both study conditions will be assessed and compared. In addition, the bracelet’s ability (on top of the daily symptom diary data) to detect early or asymptomatic infections will be assessed within all subjects. This analysis will allow subjects to serve as their own controls, testing the potential efficacy of the app plus bracelet over and above the effects of symptom reporting alone. In conclusion, this project will deliver a large body of information on SARS-CoV-2 and COVID-19 prevalence and incidence in addition to validating remote vital signs and self-reported symptom monitoring systems for early infection detection.

# OBJECTIVES

## Primary Objectives:

Using laboratory-confirmed SARS-CoV-2 infections (detected via serology tests, PCR tests and/or antigen tests) as the gold standard, we will determine the sensitivity, specificity, positive predictive value (PPV) and negative predictive value (NPV) for each of the following two algorithms to detect *first-time* SARS-CoV-2 infection including early or asymptomatic infection: the algorithm using Ava Bracelet data when coupled with the self-reported Daily Symptom Diary data, and the algorithm using self-reported Daily Symptom Diary data alone. In addition, we will determine which of the two algorithms has superior performance characteristics for detecting SARS-CoV-2 infection including early or asymptomatic infection as confirmed by SARS-CoV-2 virus testing.

## Secondary Objective(s):

1. To assess the health economic utilization of a wearable device and mobile application as measured by endpoints such as:
   1. Number of tests for suspected SARS-CoV-2 infections
   2. Number of hospitalizations
   3. Number of ICU events
   4. Number of days requiring breathing support (e.g. oxygen and/or ventilation)
2. To determine the incidence of SARS-CoV-2 infection during the study
3. Identify which features of the Ava bracelet and Daily Symptom Diary are the most predictive features
4. Assess the independent predictiveness of the Ava bracelet without use of the self-reported daily symptom data
5. To assess the time from algorithm indication to symptom onset in the subject
6. To assess the time from algorithm indication to testing of the subject
7. To determine how taking antipyretic medication affects physiological parameters among subjects using the Ava bracelet and Daily Symptom Diary
8. To determine how self-reported vaccinations (other than for COVID-19) cause changes in biophysical parameters from subject’s given baseline measurements
9. To correlate the longitudinal symptoms reported by study subjects confirmed to have COVID-19 with changes over time in their physiological data
10. To characterise longitudinal symptoms and recovery dynamics reported by the study subjects to the presence or absence of lab-confirmed SARS-CoV-2 infection
11. To assess the adherence to daily symptom diary completion and Ava bracelet wearing and synchronising over time
12. To assess the impact of the Ava bracelet on adherence to daily symptom diary completion (duration of usage and percentage of days entered)
13. To determine the time necessary to establish baseline physiological parameters per subject in the Learning Phase of the study
14. To determine if retrospective identification of the moment of potential SARS-CoV-2 infection is possible for subjects who are seropositive at the end of a period but were negative at baseline and did not receive an alert to get tested during the study
15. To determine how self-reported COVID-19 vaccinations cause changes in biophysical parameters from subject’s given baseline measurements
16. To determine the rate of breakthrough and asymptomatic COVID-19 infections among vaccinated individuals
17. To assess the influence of self-reported COVID-19 vaccinations on compliance to study procedures

## Exploratory Objective(s):

1. To assess how vaccination by different vaccines and across time affects the trajectory of SARS-CoV-2 infection
2. To determine whether other general, non-COVID-19 respiratory infections (as measured by self-reported symptoms and a negative diagnostic or antibody test for COVID-19) can be detected earlier by changes in physiological parameters
3. To determine whether COVID-19 differs from other respiratory illnesses in biophysical changes from baseline during the pre-symptomatic period
4. To determine whether women using hormonal contraception (HC) are less likely to contract SARS-COV-2 than menstruating women not on HC or menstruating women on non-HC
5. To determine whether normally menstruating women not on HC are more susceptible to infection by SARS-CoV-2 based on where they are in their menstrual cycles

# STUDY DESIGN

## Design

COVID-RED is a randomized, single-blinded, two-period, two-sequence crossover trial. 20,000 subjects will be recruited, randomized 1:1 to either Sequence 1 (experimental condition followed by control condition) or Sequence 2 (control condition followed by experimental condition). Subjects participate in a Learning Phase (2 weeks to 3 months), Period 1 (3 months), and Period 2 (3 months) (Table 1). All subjects will be instructed to log symptoms in the Daily Symptom Diary within the Ava COVID-RED app, and wear the Ava bracelet each night and synchronise it with the app each following day, throughout the full period (up to 9 months) of their study participation. In the Learning Phase baseline physiological parameters of each subject will be determined and data will be collected for further algorithm development. Subjects will receive real-time recommendations (for example, to get tested) based on data from the Daily Symptom Diary and/or physiological data from the Ava bracelet. Subjects will then move into Period 1, followed by Period 2, and will be unaware whether the feedback they receive during these periods is based on the Daily Symptom Diary data alone (control condition) or based on the diary and bracelet data (experimental condition). Based on demographics, medical history and/or profession, each subject will be stratified at baseline into a high-risk and normal-risk group within each sequence. This results in approximately 6,500 normal-risk individuals and 3,500 high-risk individuals per sequence. See Table 1 for a tabular representation of this design.

Table 1 Crossover trial design for COVID-RED

|  | **Learning Phase**  (up to 3 months) | **Period 1** (3 months) | **Period 2**  (3 months) | |
| --- | --- | --- | --- | --- |
| **Sequence 1**  (6,500 normal-risk & 3,500 high-risk) | Algorithm training & baselining | Experimental Condition | Control  Condition | |
|  |  |  |  |  |
| **Sequence 2** (6,500 normal-risk & 3,500 high-risk) |  | Control  Condition | Experimental Condition | |

## Data collection

The primary objective of this study is to evaluate the ability to detect SARS-CoV-2 infections using the Ava bracelet when coupled with the Daily Symptom Diary using the Ava COVID-RED app as compared to a control condition in which data from the Ava bracelet is disregarded in the applied algorithm. This is a reasonable control condition choice to assess an incremental gain. As such, two main methodologies will be used to obtain relevant data from subjects throughout the study to assess the primary objective:

- The Ava bracelet will track daily changes in wrist skin temperature, respiratory rate, and pulse rate. While subjects will wear and synchronise this data throughout the study, data from the bracelet will inform the applied machine learning algorithm only in the experimental condition and in the Learning Phase of the study.
- The Daily Symptom Diary included in the Ava COVID-RED app allows subjects to self-report daily on experienced symptoms and other relevant information (e.g., alcohol consumption). Data from the Daily Symptom Diary will always inform the applied algorithms throughout the study.

## Implementation and development of algorithms

For both the control and experimental conditions, separate algorithms will be applied. For the control condition, an algorithm meant to predict potential active SARS-CoV-2 infections will be applied that incorporates deterministic expert-based rules regarding the presence of SARS-CoV-2 symptoms defined by the Dutch national institute for public health and the environment ‘Rijksinstituut voor Volksgezondheid en Milieu’ (RIVM) guidelines (at the time of writing these include: common cold symptoms [such as a nasal congestion, runny nose, sneezing, sore throat], coughing, shortness of breath, elevated temperature or fever, and sudden loss of smell and/or taste [without nasal congestion]). Any subject that experiences any of the above symptoms will be advised to get tested. For the experimental condition, a machine learning algorithm developed for the same purpose based on both symptom and confounder data as well as physiological parameters collected through the Ava bracelet will be applied on data collected during the study from the Daily Symptom Diary and the Ava bracelet. Both algorithms will give recommendations to the subjects whether they should get tested for SARS-CoV-2 infection. For the algorithm in the experimental condition to give a recommendation based on physiological data, it is first required to establish a baseline per subject of their “normal” physiological patterns. The period of time in which this baseline is determined is referred to as “baselining”. This will take place at the beginning of the Learning Phase of the study and will not be repeated within each period of the crossover portion of the trial. As soon as a stable baseline has been determined for all measured physiological parameters, the algorithm can start detecting when there are significant deviations from this baseline. For the remainder of the Learning Phase the experimental condition algorithm will be applied. In Period 1 and 2, data from only the Daily Symptom Diary (control condition) or both the Diary and the bracelet (experimental condition) will be used to inform the algorithms that give indications to the subjects whether they should seek testing.

In the Learning Phase and Period 1 of the study, versions of the algorithms will be applied that have been developed and trained using data accrued during an ongoing feasibility study in Liechtenstein (COVI GAPP study) and proprietary historical data from Ava Fertility Tracker bracelet users. These will be collectively referred to as the version 1 (V1) algorithms. Data collected during the Learning Phase of this study will be used to update and inform the algorithms which will then be implemented in Period 2. These versions of the algorithms will be collectively referred to as the version 2 (V2) algorithms. As such, Period 1 and 2 will evaluate different versions of the algorithms and provide insight into the possible improvement of the algorithms over time due to the increased availability of data for development. The applied version of the algorithms at different moments in the study will be taken into account during the statistical analysis. The final version of the algorithm (version 3; V3), which will be developed using all the data collected during the study, will also be applied retrospectively to all subjects, thereby enabling investigation of how the algorithm performed as compared to earlier versions. See Figure 2 for an illustration of this process.

## Algorithm indicators

In both study conditions, the respective algorithms will alert subjects about their real-time health status in the Ava COVID-RED app through 3 types of indicators:

- **Green light:** No deviations in physical health (symptoms or biophysical parameters) detected. No recommendation is given to the subjects.
- **Yellow light:** Subjects are experiencing some symptoms but not those strictly defined as related to COVID-19 or biophysical parameters deviate slightly from baseline but do not meet the threshold value for a Red light indicator. The Yellow light indicator asks participants to consider self-isolating and keeping an eye on the Ava COVID-RED app the following day.
- **Red light:** Significant changes in biophysical parameters have been detected or symptoms that meet the RIVM criteria have been reported. The Red light indicator suggests subjects consider getting tested for COVID-19 and/or to consult a medical professional.

In all cases of a red-light indicator, subjects will receive the following generic message on the home screen of the Ava COVID-RED app after synchronising their bracelet or logging their symptoms within the last 24 hours:

“Your data suggests changes in physiological parameters and/or symptoms that might be related to a potential COVID-19 infection. Please consider seeking testing and/or input from a medical professional.”

When subjects obtain a positive PCR/antigen or serology test result during the study, they will continue to be in the study but will be moved into a so-called “COVID-positive” mode in the Ava COVID-RED app. This means that they will no longer receive recommendations from the algorithms but can still contribute and track symptom and bracelet data.

## SARS-CoV-2 infection testing

This study will use the existing testing infrastructure in the Netherlands provided by the Municipal Health services (GGD). At the time of writing, any citizen of the Netherlands has access to free polymerase chain reaction (PCR) or antigen testing for SARS-CoV-2 if they experience any of the specific symptoms defined by the RIVM guidelines (at the time of writing, these include: common cold symptoms [such as a nasal cold, runny nose, sneezing, sore throat], coughing, shortness of breath, elevated temperature or fever, and sudden loss of smell and/or taste [without nasal congestion]). As per 1 December 2020, asymptomatic persons who have been in close contact with someone who tested SARS-CoV-2-positive (as determined by the GGD contact-tracing program or by the CoronaMelder app) will also qualify for testing. SARS-CoV-2 virus testing procedures at the GGD (types of tests and criteria for testing) may change during the study period; up-to-date testing guidelines are always available on the RIVM website; https://www.rivm.nl/. During the study, subjects will be instructed to use GGD testing where possible and appropriate because this will automatically enter them into the national contact-tracing program. The results of the GGD test will be communicated to the subject directly through a web portal or by phone, as is done on a national level for every individual who gets tested. Changes in national testing policy and the types of tests used regionally will be monitored and taken into account where appropriate.

All subjects who receive a red light indication to get testing will be instructed to first contact the GGD for testing. If they do not satisfy the criteria for GGD testing, subjects can use nasal swab sampling kits provided by the study team to collect a sample themselves which they will send to the central study laboratory for testing using provided return envelopes. Subjects will be asked to log all SARS-CoV-2 test results into the Ava COVID-RED app regardless of where the testing was done. Additional details surrounding the location, accessibility and types of tests will be requested through periodic surveys. The study team will report all subjects who tested positive to the GGD (SARS-CoV-2 is a notifiable disease in the Netherlands) so that the GGD can initiate contact-tracing.

## Serology testing for SARS-CoV-2

Subjects will also be asked to donate fingerprick blood for SARS-CoV-2 serology (antibody) testing at baseline, at the end of the Learning Phase, and at the end of each period to determine if and when (during which period) they became infected with SARS-CoV-2. Baseline samples of subjects will only be analysed if the sample collected at the end of the Learning Phase is positive for SARS-CoV-2 antibodies. Samples of subjects collected at the end of Period 1 will only be analysed if the sample collected at the end of Period 2 is positive for SARS-CoV-2 antibodies.

## COVID-19 Vaccines

As of December 2020, the first of likely several vaccines developed specifically for COVID-19 have been approved by European regulatory authorities (EMA). Like many other countries the government of the Netherlands is aiming to vaccinate the majority of its population by the end of 2021. Given the differences in effectiveness between manufactured vaccines, the uncertainties surrounding the duration of protection afforded by these vaccines, as well as the difficulty in vaccinating a large majority of the population in a timely manner, the (early) detection of COVID-19 remains a crucial component to contain the pandemic in the future. While vaccinated individuals will not be eligible for this study, it is expected that a portion of the sample will be vaccinated during follow-up. Subjects are asked in the bi-weekly surveys over the course of their participation in the study whether they have received a COVID-19 vaccine in the preceding two weeks and, if so, which one (see [Section 3.8](#_Additional_outcome_measures)). Subjects that have been vaccinated will be asked to remain in the study and continue to follow the study procedures. They will continue to receive notifications from the Ava COVID-RED app depending on the condition that they are in and will be asked to keep getting tested when requested seeing as COVID-19 vaccinations are not expected to provide 100% protection.

## Additional outcome measures

Additional outcome measures collected outside the Ava COVID-RED app will include:

1. Baseline demographic and risk factors (collected using an online form at subject registration)
2. Epidemiological and infection risk information (collected using bi‑weekly online surveys)
3. Self-reported data on time to testing, testing procedures and the results (collected using bi-weekly online surveys)
4. Self-reported data on reception of COVID-19 vaccination (collected using bi-weekly online surveys)
5. Medical event reporting by subjects who have interactions with a general practitioner or a hospital related to SARS-CoV-2 infection, providing healthcare utilization measures during follow-up (collected using bi-weekly online surveys and followed-up by structured phone interviews)
6. Adverse device effects reporting by subjects (collected using bi-weekly online surveys and followed-up by structured phone interviews)

See Figure 1 for an overview of the study design described above. See Figure 2 for an illustration of the planning and logistics of the trial as described above. Note that this is a schematic representation and may not fully reflect the exact conduct of the trial.


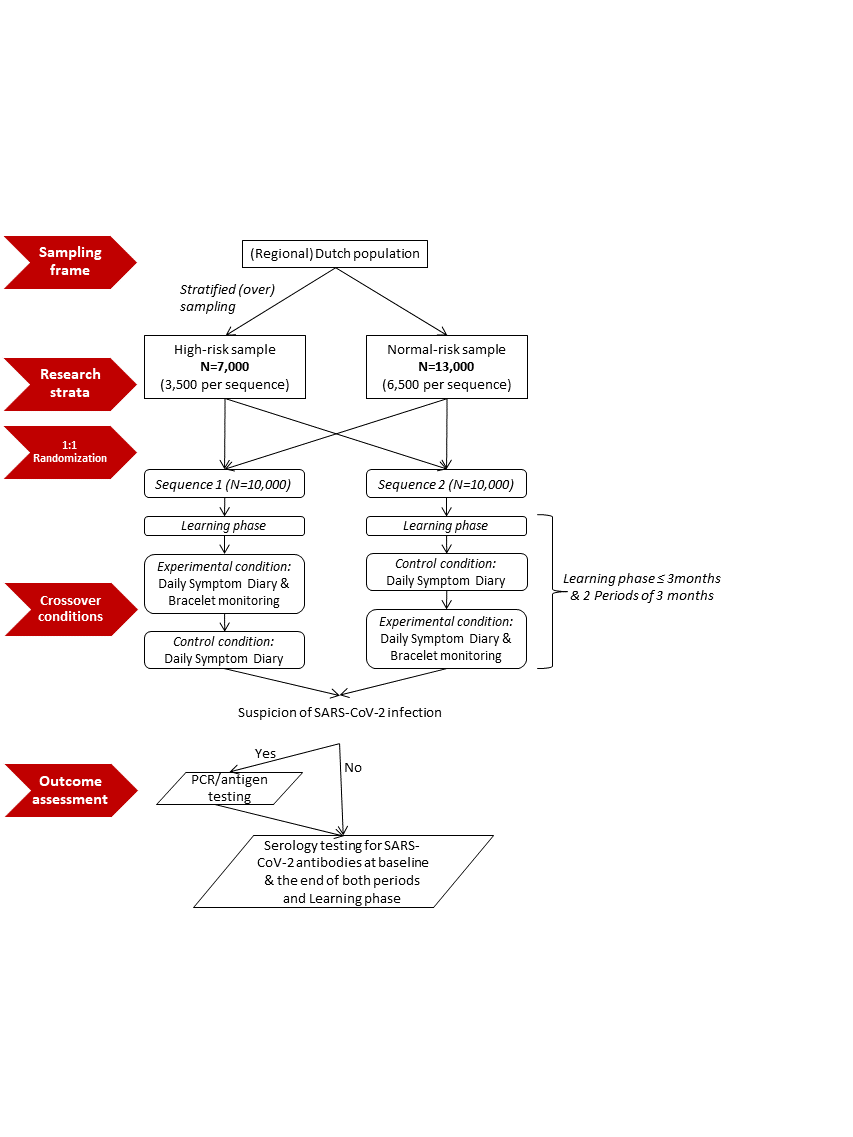


Figure 1: Study design of COVID-RED


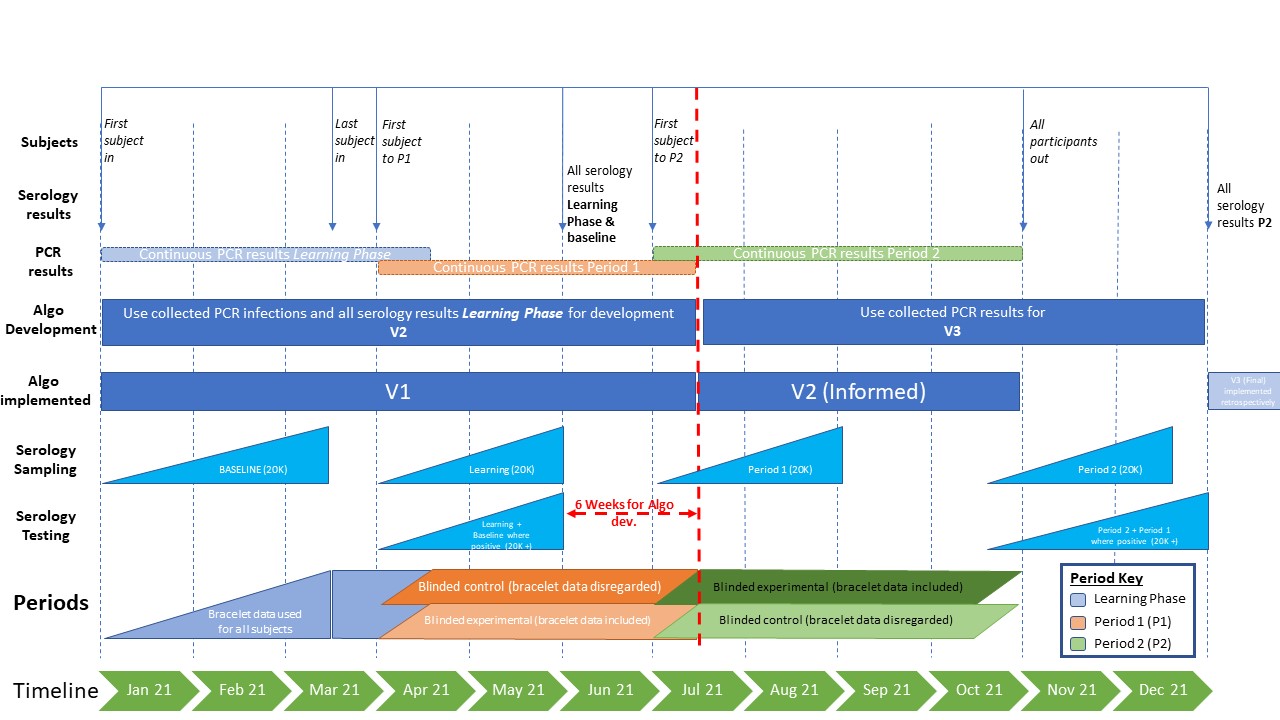


Figure 2: Schematic illustration of the planning and procedures of the COVID-RED trial

# STUDY POPULATION

## Population (base)

A target of 20,000 subjects will be enrolled in this study. Subjects will initially be recruited from existing cohorts as well as via public campaigns. Members of existing cohorts will be contacted through email or mail to inform them about the COVID-RED study and to invite them to visit the COVID-RED web portal. For the public campaign, a public link will be available for any individual who wants to know more about the study and would like to participate. Subjects visit the COVID-RED web portal where it will be verified if the subject meets the criteria to join the study and information on participation in the study is provided. When the subject is interested to join the study the consent can be given in the portal. More details on the recruitment and consent process are included in section 8 and 11.2.

## Inclusion criteria

In order to be eligible to participate in this study, a subject must meet all of the following criteria:

1. Resident of the Netherlands
2. At least 18 years old
3. Informed consent provided (electronic)
4. Willing to adhere to the study procedures described in this protocol
5. Must have a smartphone that runs at least Android 8.0 or iOS 13.0 operating systems and is active for the duration of the study (in the case of a change of mobile number, study team should be notified)
6. Be able to read, understand and write Dutch

## Exclusion criteria

A potential subject who meets any of the following criteria will be excluded from participation in this study:

1. Previous positive SARS-CoV-2 test result (confirmed either through PCR/antigen or antibody tests) (self-reported)
2. Previously received a vaccine developed specifically for COVID-19 or in possession of an appointment for vaccination in the near future
3. Current suspected (e.g. waiting for test result) coronavirus infection or symptoms of a coronavirus infection (self-reported)
4. Participating in any other COVID-19 clinical drug, vaccine, or medical device trial
5. Electronic implanted device (such as a pacemaker)
6. Pregnant at time of informed consent (self-reported)
7. Suffering from cholinergic urticaria (per the Ava bracelet’s User Manual)
8. Staff involved in the management or conduct of this study

Individuals with tendonitis, carpal tunnel syndrome, or other musculoskeletal disorders should consult their doctor prior to using the Ava bracelet.

When the informed consent is provided in the portal (see sections 8 and 11), some baseline questions will be asked after which the subject will be randomized and receive the starter kit (Section 8.4.6).

## Sample size calculation

A crossover design will be used in the current study meaning that subjects will contribute data to both the control and experimental condition. Unlike a traditional crossover study, however, the proposed crossover design includes a missing-data mechanism by design. Subjects with SARS-CoV-2 infection in the Learning Phase will have their data excluded from Period 1 and Period 2. Likewise, subjects with SARS-CoV-2 infection in Period 1 will have their data excluded from Period 2. This prevents the application of standard statistical adjustments due to missing-data. A bootstrap approach will be used to conduct statistical testing which accounts for this missing data mechanism in those cases when both periods are taken into account in the analysis. For more details, refer to Section 10 (Statistical analysis). With analyses based on bootstrap methods, classical power calculations are not possible. However, we can gain an indication of the bounds on power that can be expected. Additionally, power is highly dependent on the infection rate in the Netherlands during the conduct of the trial and for which we expect continued volatility, therefore we present power approximations based on the data reported by the RIVM on the number of individuals tested and found positive for SARS-CoV-2 infection in Weeks 36 through 40 of 2020, see Table 2.

Table 2: Number of individuals tested in the Netherlands and amount of positive tests as reported by RIVM in Weeks 36 through 40 of 2020

|  | Week 36 | Week 37 | Week 38 | Week 39 | Week 40 |
| --- | --- | --- | --- | --- | --- |
| Individuals Tested | 180,182 | 191,204 | 206,670 | 207,670 | 223,274 |
| Positive tests | 5,427 | 7,640 | 12,217 | 16,602 | 23,264 |

*.*

### Ever-infected analysis

The first analysis that will be performed for the primary analysis, a Cochrane-Mantel-Haenszel test, can be approximated by a Fisher exact test and we will use its corresponding power calculation. We provide power estimates based upon 10,000 subjects per arm as will approximately be available within Period 2. In Table 3 and Table 4 both scenarios are presented given different assumed infection rates in the Netherlands and for various differences in sensitivity (Table 3) and specificity (Table 4) to be detected. From these assessments of the trial power, we see that the most recent RIVM data lead to sufficient power for differences in sensitivity of at least 5.5%. We also see that the trial is well powered for very small changes in specificity with sufficient power for differences of at least 1.5%.

Table 3: Trial power for a Fisher exact test to detect differences in sensitivity based on infections rates of Weeks 36 through 40 of 2020

|  | Week 36 | Week 37 | Week 38 | Week 39 | Week 40 |
| --- | --- | --- | --- | --- | --- |
|  | 10,000 | 10,000 | 10,000 | 10,000 | 10,000 |
| Difference in Sensitivity 1% | 0.03 | 0.03 | 0.04 | 0.04 | 0.05 |
| Difference in Sensitivity 2.5% | 0.03 | 0.05 | 0.07 | 0.09 | 0.13 |
| Difference in Sensitivity 4% | 0.06 | 0.1 | 0.16 | 0.23 | 0.33 |
| Difference in Sensitivity 5.5% | 0.1 | 0.18 | 0.31 | 0.45 | 0.62 |
| Difference in Sensitivity 7% | 0.18 | 0.32 | 0.55 | 0.72 | 0.88 |

Table 4: Trial power for a Fisher exact test to detect differences in sensitivity based on infection rates of Weeks 36 through 40 of 2020

|  | Week 36 | Week 37 | Week 38 | Week 39 | Week 40 |
| --- | --- | --- | --- | --- | --- |
|  | 10,000 | 10,000 | 10,000 | 10,000 | 10,000 |
| Difference in Specificity  -3% | 1 | 1 | 1 | 1 | 1 |
| Difference in Specificity  -1.5% | 0.79 | 0.78 | 0.75 | 0.76 | 0.73 |
| Difference in Specificity 1.5% | 0.82 | 0.79 | 0.77 | 0.76 | 0.76 |
| Difference in Specificity 3% | 1 | 1 | 1 | 1 | 1 |

### Early detection analysis

The second statistical analysis that will be performed for the primary objective concerns the timing of each study condition to detect SARS-CoV-2. The time to first SARS-CoV-2 indication or infection will thus be compared between the conditions. This will be tested using a stratified log-rank test. Frequently, power calculations for the log-rank test assume some form of proportional hazards or a specific parametric family for the study arms. In this study, we expect the experimental condition to be able to detect SARS-CoV-2 infection earlier due to the possible detection of pre- or asymptomatic individuals due to deviations in physiological parameters. However, when subjects become symptomatic, the algorithms in both the control and experimental condition will each declare subjects to likely be infected at the same moment by definition. To account for this aspect, a piecewise log-rank test power calculation is used to characterize the trial power. The assumption in the piece-wise hazards is that initially the experimental condition will have a higher chance to detect SARS-CoV-2 infection; however, this difference will decrease over time resulting in equal hazards in the later pieces of the hazard function. As described in the first analysis above, the proposed crossover design results in an application of bootstrap statistical testing so that the power calculations in Table 5 are rough approximations. The estimated power is bounded below by a parallel-arm study with 10,000 subjects per condition and above by a study with 20,000 subjects per condition. From these bounds it appears that the trial is well powered under all of the recently reported RIVM data.

Table 5: Lower (10,000) and upper (20,000 ) bounds of the trial power for a piecewise log-rank test to detect differences between study conditions on the time to first SARS-CoV-2 indication or infection based on infection rates of Weeks 36 through 40 of 2020

|  | Week 36 | | Week 37 | | Week 38 | | Week 39 | | Week 40 | |
| --- | --- | --- | --- | --- | --- | --- | --- | --- | --- | --- |
|  | 10k | 20k | 10k | 20k | 10k | 20k | 10k | 20k | 10k | 20k |
| Power | 0.66 | 0.92 | 0.80 | 0.98 | 0.94 | 0.99 | 0.99 | 1.00 | 1.00 | 1.00 |
|  |  |  |  |  |  |  |  |  |  |  |

# TREATMENT OF SUBJECTS

This interventional study will involve an experimental device, experimental app and algorithms, and the collection of blood and/or mucus for testing with approved laboratory tests.

## Investigational product/treatment

Subjects will be randomly assigned to one of two sequences in which they will receive the experimental and control condition in random order. In the control condition, only data of the Daily Symptom Diary will be included in the feedback to the patient about their potential infection status; and in the experimental condition, both data of the Daily Symptom Diary and the Ava bracelet will be included in the feedback to the patient about their potential infection status. Subjects will be instructed to log symptom data in the Ava COVID-RED app (Daily Symptom Dairy) every day and wear their Ava bracelet throughout the study nightly while they sleep. Upon waking, they will synchronise the Ava bracelet to the corresponding Ava COVID-RED app on their smartphone. Subjects will receive real-time feedback in-app about their symptoms and in line with current Dutch national testing standards they should seek testing for SARS CoV2. Subjects will be blinded, which means that they will not know if the real time feedback about their potential infections status will come solely from the Daily Symptom Diary, or if this feedback is based on data from both the Daily Symptom Diary and the Ava bracelet.

## Use of co-intervention (if applicable)

Not applicable.

## Escape medication (if applicable)

Not applicable.

# INVESTIGATIONAL PRODUCT

#

## Name and description of investigational product(s)

Ava COVID-RED is an investigational product developed specifically for the COVID-RED clinical trial. The device uses machine learning and wearable sensor technology to help users monitor their general health and well-being. In particular, its algorithms are fine-tuned to detect changes in physiological and/or self-reported symptoms that could indicate a potential SARS-CoV-2 infection. Ava COVID-RED is a non-invasive device made up of the following components as illustrated in Figure 3 below:

- *The Ava Bracelet incorporating hardware with electronics and embedded software (Hardware Bracelet Generation 2.0)*
- *A mobile application running on smartphones (iOS and Android)*
- *Backend software, including the COVID-19 infection monitoring algorithm, running on remote servers.*

The wrist-worn bracelet acts as a data logger, recording and storing user’s physiological sensor signals as raw datasets throughout the night. The user synchronizes the Ava bracelet to the mobile application (AVA COVID-RED app) the following morning. The mobile app reads out all the raw datasets via Bluetooth Low Energy (BLE) and transfers them to the backend. After computation and pre-processing of the physiological parameters based on the bracelet recordings, the COVID-19 infection monitoring algorithm is run. The obtained pre-processed physiological parameters and potential COVID-19 infection indications are transferred back to the mobile app and displayed to the user.


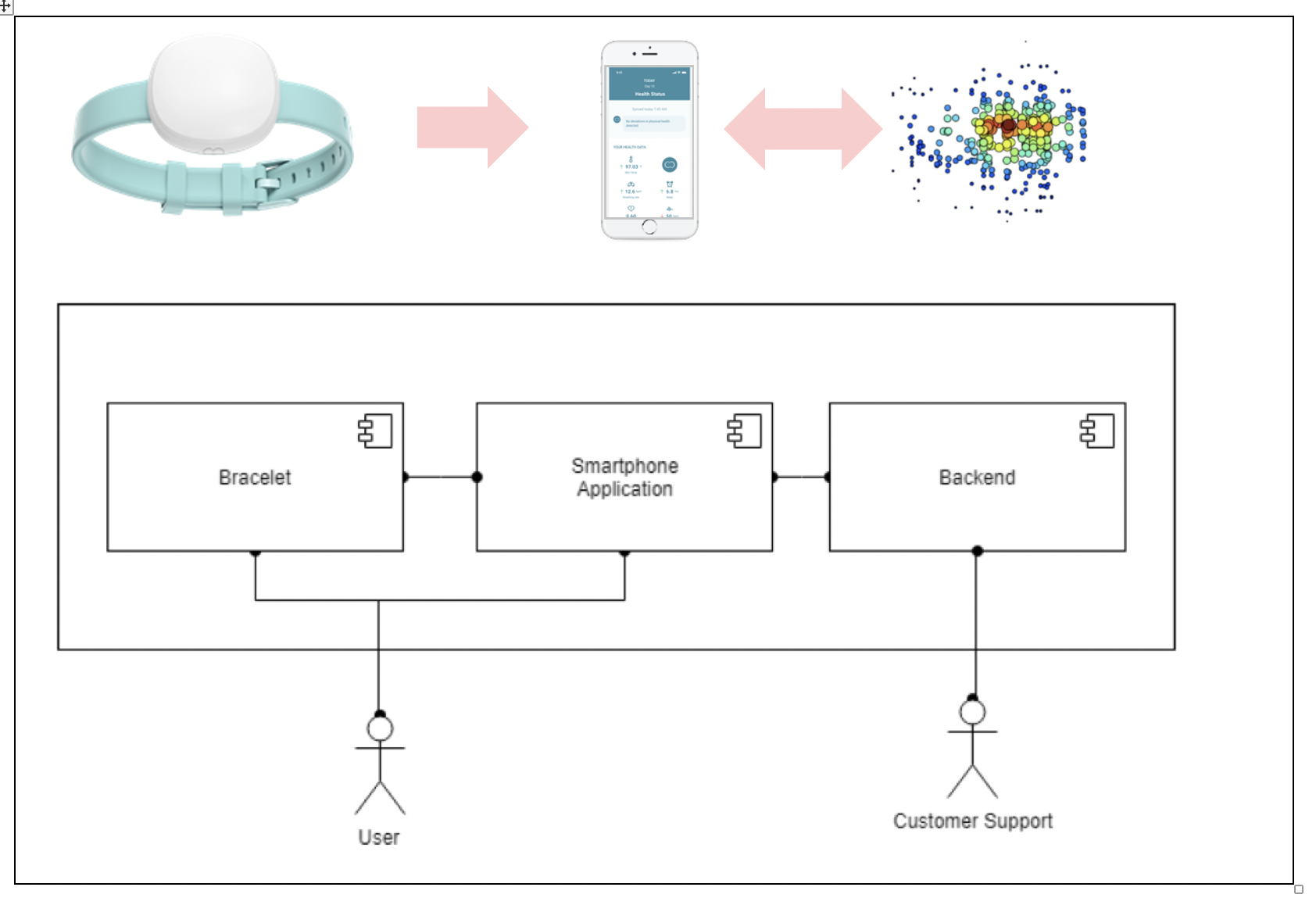


Figure 3: Ava-COVID- Visual of Components

The AVA COVID app is also utilized to collect the Daily Symptom Diary data. The user completes The Daily Symptom Diary in the app, and they will report any symptoms they may be experiencing. The app transfers this information to the backend server and the COVID-19 infection monitoring algorithm is run on the self-reported Daily Symptom Diary data only or in combination with the bracelet data depending on condition. Potential COVID-19 infection indications are transferred back to the mobile app and displayed to the user.

Ava COVID-RED is based on a fertility-related Conformité Européenne (CE) marked predecessor (Ava Fertility Tracker); its codebase was adapted from the Fertility Tracker software, while its hardware component is identical to its predecessor. Along with the hardware, the method of physiological data collection for Ava COVID-RED remains unchanged from its predecessor. The underlying algorithm, however, has been updated to provide a health indicator as opposed to a prediction of the user’s fertile window. The Ava Fertility Tracker is still on the market in Europe and North America.

Ava COVID-RED was developed for clinical research purposes only and is not currently on the market in Europe or other countries. The legal manufacturer of the Ava COVID-RED device is Ava AG, Gutstrasse 73, 8055 Zurich, Switzerland.

## Summary of findings from non-clinical studies

Results of non-clinical studies assessing the device’s raw materials and biocompatibility is discussed in Section 6.1 of the most recent version of the Investigational Medical Device Dossier (IMDD). No new biocompatibility testing was conducted for Ava COVID-RED as it used the same underlying hardware as its CE marked predecessor, the Ava Fertility Tracker. Test reports related to the external validation of hardware materials have been appended to the IMDD and include:

- Cytotoxicity Test Report
- Biological Risk Assessment
- Sensitization Test
- Skin Irritation Test Report

Additional non-clinical study data includes findings from a thorough literature evaluation related to the safety profile of wearable devices and COVID-19 detection, documented in Section 6.1.a of the IMDD and the Ava COVID-RED Clinical Evaluation Plan. A summary of the search protocol and its findings can be found in the Ava COVID-RED Clinical Evaluation Plan.

## Summary of findings from clinical studies

Results from clinical studies establishing the efficacy of Ava-COVID’s predecessor device, including the shared underlying hardware, are described in the Ava COVID-RED Clinical Evaluation Plan. The Clinical Evaluation Plan also outlines the exploratory data analysis which will drive the algorithm development for Ava-COVID. The Clinical Evaluation Plan discusses the clinical evaluation of Ava-COVID, identifying its role in the COVID-RED and COVI-GAPP clinical trials. Findings from these two trials will contribute to the Clinical Evaluation Report, to be written in December 2021 at the end of subject follow-up.

## Summary of known and potential risks and benefits

Risk analysis for Ava COVID-RED followed the manufacturer’s internal Quality Management System (QMS) specifications. Namely, a Risk management team consisting of representatives from Product, Clinical Development, Quality Affairs, Regulatory, and Software departments. The Risk management team considered both the probability and severity of harm occurring from potential hazards generated by the investigational device. A detailed description of the risk assessment process is outlined in the IMDD, with results from the investigation provided in the attached Risk Management File (see Risk Management Plan Ava04, Product properties and possible hazards Ava04, FMEA Ava04)

## Description and justification of route of administration and dosage

Not applicable as this is a wearable device and smartphone app.

## Dosages, dosage modifications and method of administration

Not applicable as this is a wearable device and smartphone app.

## Preparation and labelling of Investigational Device

Devices are provided in the commercial package for the Ava Fertility Tracker, consisting of a box with a package insert in English, French, Italian, Spanish, and German languages. A study specific label in the local language will be present on the box before it is handed out to study subjects, and additional study instructions in Dutch will be provided with the package.

## Device accountability

Devices will be provided by the manufacturer. Prior to handout to the subjects, the devices will be stored centrally as per the manufacturer’s instructions. The trial project team is responsible for keeping accurate records of the devices received from the manufacturer as well as the number of devices handed out to and returned by the subjects. Devices will be barcoded and scanned before they are handed out to the subject. At the study’s conclusion, the devices must be returned by the study subject to the project team and will be blocked by the manufacturer to prevent off-label use.

# NON-INVESTIGATIONAL PRODUCT

Not applicable.

# METHODS

## Study parameters/endpoints

Note that for each parameter and endpoint listed in 8.1.1 and 8.1.2 the measure will be observed in Period 1 and in Period 2. The parameters and endpoints will be statistically assessed within each period separately. See Section 10 for additional details. For the assessment schedule, see APPENDIX 1. Details on the assessments are provided in Section 8.4.

### Main study parameter/endpoint

The primary endpoint for this study for each subject is the daily indication of potential SARS-CoV-2 infection as provided by the algorithm based on data from the Daily Symptom Diary (control condition) or the algorithm based on both data from the Daily Symptom Diary and the Ava bracelet (experimental condition). This daily endpoint will be compared against actual SARS-CoV-2 test results (PCR/antigen or serology) collected at baseline, the end of the Learning Phase, and after each Period. For the primary comparison, this daily endpoint will be summarized over each crossover period per subject to determine (1) if a subject was ever judged to have a high risk for a potential SARS-CoV-2 infection, and (2) if a subject was ever confirmed to have a SARS-CoV-2 infection by PCR/antigen or serology testing. For this comparison, parameters such as sensitivity, specificity, positive predictive value, and negative predictive value will be calculated.

### Secondary study parameters/endpoints (if applicable)

Additional endpoints will be collected for analysis of the secondary objectives as listed in Section 2.

*Study condition compliance (collected with Ava COVID-RED app)*

- Duration of Ava bracelet use (amount of days between first day of synchronised data and last day of synchronised data)
- Duration of daily symptom diary use (amount of days between first day completed symptom data and last day completed symptom data)
- Compliance rate: percentage of days (as compared to duration of use) that Ava bracelet was worn and synchronised.
- Compliance rate: percentage of days (as compared to duration of use) that symptoms were entered into the Daily Symptom Diary of the Ava COVID-RED app

*Longitudinal characteristics of study population (collected with Ava COVID-RED app)*

- Number/percentage of non-antipyretic and antipyretic medication days
- Number and type of vaccination events
- Number/percentage of days with alcohol consumption
- Number/percentage of days with recreational drug usage
- Number/percentage of days with period (menstruating women only)
- Number of pregnancy events (menstruating women only)

*Overall incidence of SARS-CoV-2 (collected through self-reporting of PCR/antigen test results in the Ava COVID-RED app and serology results)*

- SARS-CoV-2 cumulative incidence by condition and by stratum

*Healthcare Resource Utilization (collected throughout the study through bi-weekly surveys in COVID-RED web portal, structured interviews, and Ava COVID-RED app)*

- Number of COVID-related contacts from subjects to their general practitioner (GP)
- Number and duration of COVID-related hospitalization episodes
- Number and duration of COVID-related intensive care unit (ICU) episodes
- Number and duration of COVID-related supported breathing intervention
- Number and percentage of subjects with each reported medication used during hospitalization (WHO Drug Dictionary [WHO-DD])

*COVID-19 Testing (collected throughout the study through bi-weekly surveys in COVID-RED web portal and Ava COVID-RED app):*

- Number of tests conducted by GGD
- Number of provided PCR kits used
- Number of tests conducted due to red-light indicator of Ava COVID-RED app
- Time between first recommendation for testing and contact with GGD
- Time between first recommendation for testing and contact with study team for PCR sample kit
- Time between first recommendation for testing and GGD SARS-CoV-2 test (stratified for antigen or PCR test if applicable)
- Time between first recommendation for testing and provided PCR SARS-CoV-2 test
- Time between first recommendation for testing and GGD SARS-CoV-2 test result (stratified for antigen or PCR test if applicable)
- Time between first recommendation for testing and Central laboratory SARS-CoV-2 test result

*COVID-19 Vaccination (collected throughout the study through bi-weekly surveys in COVID-RED web portal)*

- Number of subjects who received a first and second (where relevant) dose of a COVID-19 vaccination stratified by manufacturer of the vaccine
- Time between first and second (where relevant) dose of the vaccine

*Behaviour (collected throughout the study through bi-weekly surveys in COVID-RED web portal):*

- Number of behavioural changes
- Behavioural changes made and reasons
- Number of behavioural changes (partly) due to the app or wearable
- Behavioural changes (partly) due to the app or wearable and reasons

*Daily Symptom Diary data (collected daily with Ava COVID-RED app)*

- Number and duration of each contiguous reported symptom:
  - Head:
    - Confusion
    - Difficulty concentrating
    - Dizziness
    - Fever
    - Headache
  - Nose and mouth:
    - Bluish lips or face
    - Loss of smell
    - Loss of taste
    - Nasal congestion or runny nose
  - Throat and sternum:
    - Nausea
    - Sore throat
    - Vomiting
  - Chest:
    - Chest tightness, pain or pressure
    - Dry cough
    - Shortness of breath or difficulty breathing
  - Lower back:
    - Chills or excessive sweating
    - Fatigue
    - Muscle or body aches
    - Skin impurities
  - Abdomen:
    - Abdominal pain or cramping
    - Diarrhoea
    - Stomach ache
  - General malaise
  - No symptoms to report
- Average oral temperature and number/percentage of daily deviations above 38 degrees (Celsius), when collected
- Time between first algorithm indication for testing and symptom onset

### Other study parameters

*Baseline data of study population necessary for Ava COVID-RED app algorithms (Collected at registration via the Ava COVID-RED app)*

- Date of birth
- Country of birth
- Biological sex
- Weight (kg)
- Height (cm)
- Previous COVID-19 testing history
- Risk-factors:
  - Hay fever
  - Moderate to severe asthma
  - Chronic smoker’s cough
  - Chronic lung disease
  - Diabetes requiring medication
  - Hypertension requiring medication
  - Serious heart condition
  - Chronic kidney disease requiring dialysis
  - Chronic liver disease
  - Human immunodeficiency virus (HIV)
  - Immune system deficiency or autoimmune disorder
  - Haemoglobin disorders (e.g., sickle cell disease, thalassaemia)
  - Chemo-, radio- or immunotherapy in the past 6 months
  - Use of medications that weaken the immune system (e.g., corticosteroids)
  - History of bone marrow or organ transplant
- Smoking status
- Vaccine history (past 12 months)
- Menstruation status including current Birth Control methods
- Breastfeeding status

*Baseline data of study population (Collected at registration and baseline in COVID-RED web portal)*

- Biological sex
- Country of birth
- Father country of birth
- Mother country of birth
- Age
- Height
- Weight
- Healthcare Professional (Y/N)

*COVID-19 Risk Factors (Collected at registration and baseline in COVID-RED web portal)*

- Risk factors
  - Hay fever
  - Moderate to severe asthma
  - Chronic smoker’s cough
  - Chronic lung disease
  - Diabetes requiring medication
  - Hypertension requiring medication
  - Serious heart condition
  - Chronic kidney disease requiring dialysis
  - Chronic liver disease
  - Human immunodeficiency virus (HIV)
  - Immune system deficiency or autoimmune disorder
  - Haemoglobin disorders (e.g., sickle cell disease, thalassemia)
  - Chemo-, radio- or immunotherapy in the past 6 months
  - Use of medications that weaken the immune system (e.g., corticosteroids)
  - History of bone marrow or organ transplant
- Highest educational level attained
- Household situation (e.g. total number of housemates)
- Employment details
- Use of public transport
- Use of CoronaMelder App (or similar application) and duration
- Previous SARS-CoV-2 infections
- Chronic medication use

## Randomisation, blinding and treatment allocation

This is a single-blinded study. The sponsor, Investigator and the device manufacturer will be aware of the assigned sequence, but the subject will be blinded. The randomization ratio for the study sequences will be 1:1. The randomization schedule will be stratified. Based on demographics, medical history and/or profession, each subject will be stratified at baseline into a high-risk and normal-risk group within each sequence. Within each study sequence, the normal-risk stratum will be capped in order to ensure that the high-risk stratum is fully recruited. The subject will wear the Ava bracelet and complete the Daily Symptom Diary in the Ava COVID-RED app for the full duration of the study, and they will not know if the feedback to them about their potential infection status will be only based on data they entered in the Daily Symptom Diary within the Ava COVID-RED app or based on both the data from the Daily Symptom Diary and the Ava bracelet.

## Recruitment planning

All 20,000 subjects for COVID-RED will be recruited at the start of the study in the first quarter of 2021. In section 4.1, the population from which these subjects will be recruited is detailed. Subjects will be followed for up to 3 months during the Learning Phase, 3 months during Period 1, and 3 months during Period 2, for a total of up to 9 months. In Periods 1 and 2, subjects will receive both the control and experimental condition in random order. Recruitment and follow-up will be completely remote and take place via post, email, phone and electronic web portals. In this way, risk of SARS-CoV-2 infection is minimized as much as possible for those wanting to participate in the trial and for the staff conducting the trial. See Figure 2 for an illustration of the planned recruitment timelines and study procedures.

## Study procedures

In APPENDIX 1, the study procedures for the subjects are summarized for the current protocol. Further details regarding specific procedures are provided below. Figure 4 provides an overview of the subject journey including all potential moments at which subjects can fail to register, drop out and/or fail to follow protocol.

### (E)mail invitation to participate in COVID-RED

Subjects will be recruited from existing cohorts from previous studies as well as via public outreach campaigns. Members of existing cohorts will be contacted through email or mail to inform them about the COVID-RED study and to invite them to visit the COVID-RED web portal. For the public campaign, an online URL link will be available for any individual who wants to know more about the study and would like to participate.

### Registration and verification of inclusion/exclusion criteria

In the COVID-RED web portal, subjects will be asked to register by entering their e-mail address and phone number. Subsequently, subjects will fill in a pre-screening survey in which their eligibility for the study will be confirmed. Several additional demographic details will also be asked to characterize the screened population as well as the risk factors. Subjects will be asked if they give permission to the study team to collect and use this data.

### Informed consent by subject

After the eligibility verification and confirmation of risk group (normal or high), subjects are provided with further information on the study, and if subjects wish to participate, they must sign the informed consent form. Subjects will be given the chance to contact the study team for any questions they have during this process. In case the stratum for the assigned risk group is full, subjects will be notified they are not able to join the study prior to the consent process.

The full consent process is described in more detail in section 11.2.

### Baseline survey

Subjects will be asked to enter baseline information on socio-demographic characteristics, comorbidities, medication use, and COVID-19 risk factors in the COVID-RED web portal.

### Assignment of Unique Identification code (UID) and randomization to sequence

Following the successful signing of informed consent form, subjects will be stratified based on their demographic information provided at registration to either the normal-risk or high-risk group. In addition, they will be assigned a UID and randomized to one of the two sequences. Subjects will be blinded as to the sequence that they have been assigned to.

### Receive starter kit

Subjects will then receive a starter kit through the postal service. This starter kit will contain the following items: Ava bracelet, self-sampling kits for serology tests, detailed instructions on how to use the self-sampling kits, general study instructions for the Ava bracelet and Ava COVID-RED app and pre-addressed (medical) return envelopes with prepaid postage.

### Installation of the Ava COVID-RED app, log in and pairing of Ava bracelet

Subjects will receive their login details along with instructions on how to download the Ava COVID-RED app from the iOS and Android app stores on their smartphone. Subjects will be asked to install the app and log in using their credentials. After logging in, subjects will be able to change their password by navigating to the My Profile part of the Ava COVID-RED app; instructions for how to reset their password will be provided as part of the starter kit. Subjects will need to provide a personal email address within the Ava COVID-RED app to enable password recovery features. The personal email address will not be viewable by trial staff including Ava employees. At first login, subjects will also complete a survey in-app containing basic demographic and epidemiological information which will be necessary for the development and implementation of the algorithms. All subjects will also be instructed to pair the received Ava bracelet with the Ava COVID-RED app prior to wearing it for the first time.

### Collection & testing of plasma/blood sample

Subjects will be asked to self-collect a capillary blood sample using provided materials at Baseline as well as upon completion of the Learning Phase, Period 1 and Period 2 by fingerprick for a total of 4 samples during the study. Subjects will be requested to send the sample to the central laboratory within 24 hours using provided, pre-addressed medical envelopes with prepaid postage. The samples will be processed and stored at the central laboratory on the day of receipt. All samples collected at the end of the Learning Phase, and Period 2 will directly be tested for SARS-CoV-2 antibodies using an in-house ELISA method upon receipt. By testing at the end of each stage of the study, we have nearly 100% knowledge of infection status at the end of each trial period, thereby allowing assessment of sensitivity and specificity. Baseline samples will only be analyzed if the sample collected at the end of the Learning Phase is positive for SARS-CoV-2 antibodies. Samples collected at the end of Period 1 will only be analyzed if the sample collected at the end of Period 2 is positive for SARS-CoV-2 antibodies. In this way, subjects who had a SARS-CoV-2 infection prior to participation in the study or at start of Period 2 can be identified. The central laboratory in-house ELISA method assesses total SARS-CoV-2 antibodies (Ab), and if positive, isotype-specific (IgM and IgG) SARS-CoV-2 antibodies. Subjects will be informed on their testing result for SARS-CoV-2 antibodies.

### Daily logging of symptoms and confounders in Ava COVID-RED app

Subjects will be asked to record any experienced symptoms and potential confounders (e.g., alcohol consumption) daily within the Ava COVID-RED app. Symptoms can be recorded in-app for up to 7 days after each date.

### Nightly wearing & routine syncing of the Ava bracelet

Subjects will be instructed to wear the Ava bracelet each night and synchronise it with the Ava COVID-RED app each following day for the complete follow-up. Throughout the study, subjects will be able to observe and track changes in their resting heart rate, breathing rate, skin temperature, sleep, and heart rate variability as recorded by their Ava bracelet.

### Receive algorithm-based health indicator

During follow-up, subjects will be informed by the Ava COVID-RED app whether the algorithm has detected symptoms and/or changes in physiological parameters potentially indicative of a COVID-19 infection. Following onboarding instructions provided by the study team, subjects will then be expected to seek testing for SARS-CoV-2. Algorithms will be used to assess the risk of potential SARS-CoV-2 infection. In the control condition, the algorithm is solely reliant on symptom data entered by the subject during the study and background medical information collected at registration. In the experimental condition, the algorithm is additionally reliant on physiological data collected from the Ava bracelet. If subjects are given a red-light indicator (significant changes in biophysical parameters have been detected or symptoms that meet the RIVM criteria have been reported), they will be advised to seek testing for SARS-CoV-2 infection.

### Testing for presence of SARS-CoV-2 virus

Subjects will receive specific instructions at the start of the study to always contact the GGD for SARS-CoV-2 virus testing when receiving a red-light indicator and only request for self-sampling kits for the central laboratory PCR/antigen testing service when turned down for testing by the GGD in line with the criteria applicable at that moment in time.

If subjects cannot be tested through the GGD because they do not qualify (for example, they do not have symptoms and do not have proof of a recent close contact with a case),

they will be instructed to contact the study team and request a self-sampling PCR kit. The kit will be sent to them as soon as possible including instructions. Subjects will be instructed to take the sample and use the pre-addressed medical envelope with prepaid postage to send the sample to the central laboratory within 24 hours. The samples will be processed and stored at the central laboratory on the day of receipt. Results will be communicated back to the subjects and any positive results will be reported to the GGD.

### Logging any SARS-CoV-2 test results and vaccination in Ava COVID-RED app

Subjects will be instructed to log results from a SARS-CoV-2 test, independent of where the test was obtained, directly into the Ava COVID-RED app via the Daily Symptom Diary. Results from the serum antibody testing will also be provided by the central laboratory directly to the primary investigators. If a test result is negative, the subject will continue with the study procedures as planned. If the test result is positive, the Ava COVID-RED app will continue in a COVID-positive mode during which the subject will remain in the study and will still be asked to record symptom data and wear/sync the bracelet. However, the subject will not receive any further algorithm-based indicators; instead, they will see a constant message on their Ava COVID-RED app home screen suggesting they seek additional medical care if their symptoms worsen or fail to improve.

When subjects receive any kind of vaccine (including any COVID-19 vaccine), they can log this in the Ava COVID-RED app and provide details in an unstructured open-text response field following the prompt, “If you received a new vaccine within the prior 24 hours, please specify which one: [Vaccine]”. Subjects that have received a COVID-19 vaccine during follow-up will continue using the app as instructed at onboarding and should seek testing for SARS-CoV-2 when receiving the red light indicator as described above.

.

### Periodic completion of survey

Every two weeks, a survey will also be sent to obtain more specific details on the testing procedures, epidemiological, household, occupational data and behaviour of the subjects. These surveys will allow the subject to self-report details on if they took a SARS-CoV-2 virus test, where they took it, how long they waited after receiving an in-app indicator before contacting the GGD, the time until they took their own sample and sent it to the central laboratory (if applicable), the type of test they took, how long it took them to receive their test results, and the result itself. These characteristics will also give a sense of the risk for SARS-CoV-2 infection that a subject has and can reveal important epidemiological relations as detailed in the study objectives. In addition, specific structured questions will be asked to subjects if they received a COVID-19 vaccine, including which dose (i.e., first or second) and from which manufacturer.

### Regular completion of hospital episodes and adverse device events (ADEs)

Subjects will regularly be sent an electronic survey to self-report possible hospital episodes related to COVID-19 and adverse device effects (ADEs). For hospital episodes, questions on the dates and name of the facilities will be included.

### Interview with subject for Healthcare Resource Utilization (HRU) and Adverse event (AE) reporting

In the case of a hospital episode or ADE as reported in surveys by the subject, qualified medical health professionals will contact the subject directly by phone and obtain relevant information through a structured interview. Data on HRU and ADEs will be entered directly into the eCRF system. Additionally, the clinical team will report all ADEs and device deficiencies to the Ava bracelet manufacturers within 5 business days of notification from the subject. All potential or probably serious adverse device effects (SADEs) are to be reported by the clinical team to the device manufacturer within 24 hours of notification from the subject.

### Return of Ava bracelet

At the end of the study, subjects will be instructed on how to return the Ava bracelet and any other relevant study materials to the study team.


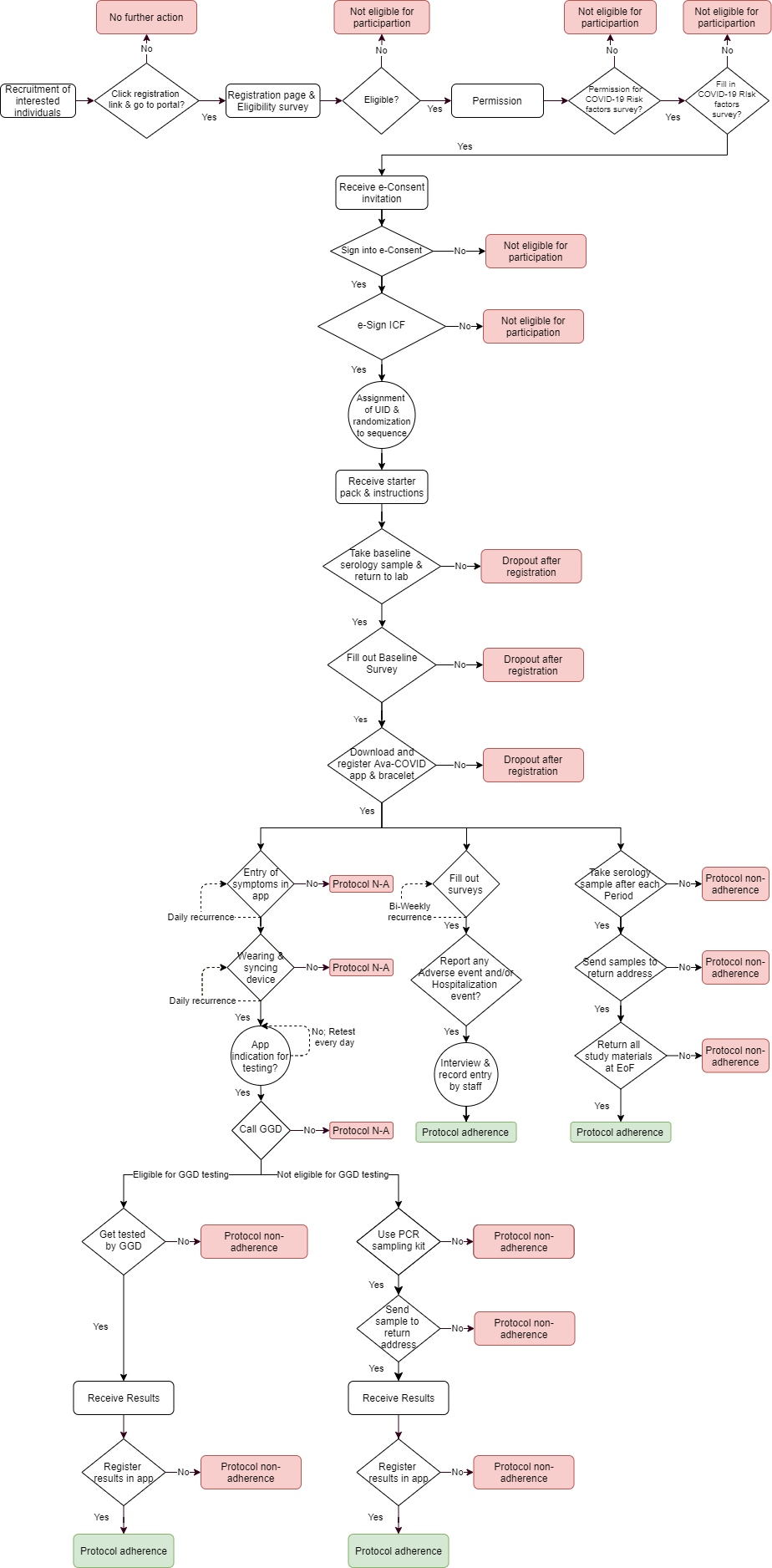


Figure 4: COVID-RED subject journey. Red shapes correspond to potential exclusion, dropout or missing information; green shapes correspond to protocol adherence.

## Withdrawal of individual subjects

Subjects can leave the study at any time for any reason if they wish to do so without any consequences. Subjects are instructed to return all trial materials to the study team when they decide to leave the study. The study team will also notify Ava within 5 business days of the participant’s withdrawal, so the device manufacturer can remotely turn off the Ava bracelet and thus ensure no off-label use occurs.

### Specific criteria for withdrawal

A subject will be withdrawn from the study for any of the following reasons:

- Lost to follow up
- Withdrawal of consent
- Death
- If a subject is poorly compliant (< 20%) with the completion of either the daily Symptom Diary or syncing of the bracelet
- If the subject is using the Ava Bracelet for other purposes as the study purpose (for example for women to detect menstrual cycle changes among ovulation)

## Replacement of individual subjects after withdrawal

There will be no replacement of subjects in this study.

## Follow-up of subjects withdrawn from treatment

To ensure the number of subjects lost to follow-up is kept to a minimum, the subject will be asked to provide contact details of a back-up person upon study entry. In the event that a subject is considered lost to follow-up, the investigator may decide to contact this back-up person to enquire if the subject is able to re-join the study. A specific study procedure will be available to support this process.

## Premature termination of the study

In case the study team, a Regulatory Authority or Independent Ethics Committee elects to terminate or suspend the study, a study-specific procedure for early termination is to be followed.

# SAFETY REPORTING

## Temporary halt for reasons of subject safety

The sponsor will suspend the study if there is sufficient ground that continuation of the study will jeopardise subject health or safety. The sponsor will notify the accredited EC without undue delay (within 2 working days and not later than 4 calendar days) of a temporary halt including the reason for such an action. The study will be suspended pending a further positive decision by the accredited EC. The investigator will take care that all subjects are kept informed.

## ADEs, SADEs and USADEs

This protocol is considered a low-risk study. The Ava bracelet is a Class IIa device for which a Declaration of Conformity is available. The hardware and sensors used to measure heart rate, breathing rate, wrist skin temperature, heart rate variability, sleep duration, and skin perfusion are identical to their counterparts in the Ava’s commercially available product, the Ava Fertility Tracker (<https://www.avawomen.com/how-ava-works/healthcare/technology/>).

The sponsor shall be responsible for documentation, assessment and reporting all safety events including adverse device effects (ADEs), severe adverse device effects (SADEs) and device deficiencies according to local and national regulatory requirements. The sponsor shall report all Ava device-related safety and device deficiency information to Ava’s customer service for vigilance purposes.

### Adverse device effects (ADEs)

Adverse events, in general, are defined as any undesirable experience occurring to a subject during the study, whether or not considered related to the device or the study. In this trial, we will restrict the recording of adverse events to only the device related events (ADEs) which will be reported. The subjects will be reminded regularly to report any occurring ADEs related to the (use of the) device through a survey. These ADEs will be followed up (see section 9.4) by the investigator and entered in the eCRF.

All ADEs will be reported to the manufacturer within 5 business days of notification from the subject.

### Serious adverse device effects (SADEs)

A serious adverse event, in general, is any untoward medical occurrence or effect that

- results in death;
- is life threatening (at the time of the event);
- requires hospitalisation or prolongation of existing inpatients’ hospitalisation;
- results in persistent or significant disability or incapacity;
- is a congenital anomaly or birth defect; or
- any other important medical event that does not result in any of the outcomes listed above due to medical or surgical intervention but could have been based upon appropriate judgement by the investigator.

An elective hospital admission will not be considered a reason to consider an event serious.

In this trial, we will restrict the recording of SAEs to only the device-related SAEs (SADEs). These will be reported to the manufacturer within 24 hours of receiving notification from the subject. Each SADE that may occur during the course of the COVID-RED clinical trial should be documented on the Spontaneous Adverse Event and Device Deficiency Report Form provided by the manufacturer. This form should also be completed for device deficiencies that could have resulted in a SADE under different circumstances. The Spontaneous Adverse Event and Device Deficiency Report Form must be completed in as much detail as possible, including at the minimum the device serial number and country of event. Whenever possible, pictures documenting the ADE, SADE and/or device deficiency should be included. The completed form should be sent to the following customer service support email address:

[support@avawomen.com](mailto:support@avawomen.com)

Seriousness is judged by the study investigator.

All serious adverse events that lead to (temporary) suspension/stop of the clinical trial or adjustment in the medical device and all unexpected serious adverse device reactions which indicate an imminent risk of death, serious injury, or serious illness and that require prompt remedial action for other patients/subjects, users or other persons, will be reported expedited to the CCMO and EC within 2 working days and not later than 4 calendar days.

All other serious adverse device effects will be reported quarterly to the CCMO and EC via the MEDDEV 2.7.3 form.

### Unanticipated serious adverse device effects (USADEs)

Serious adverse device effects are considered unanticipated serious adverse device effects (USADEs) if the following 3 conditions are met:

- The event is serious (see the definition above)
- There is a certain degree of probability that the event is a harmful and an undesirable reaction to the medical device under investigation,
- The adverse device reaction is unexpected, i.e, the nature and severity of the adverse device reaction are not in agreement with the product information as documented in the reference information (in this study: the Investigational Medical Device Dossier [IMDD])

## Annual safety report

Not applicable

## Follow-up of adverse device effects

All (S)ADEs will be followed until they have abated, or until a stable situation has been reached. Depending on the event, follow-up may require additional tests or medical procedures as indicated, and/or referral to the general physician or a medical specialist.

SADEs need to be reported until the end of study in the Netherlands.

## Data Safety Monitoring Board (DSMB) / Safety Committee

Not applicable

# STATISTICAL ANALYSIS

Below an overview is given regarding the planned statistical analyses for the trial. Further details as well as additional models and sensitivity analyses will be provided in a separate Statistical Analysis Plan.

*General statistical approach*

Appropriate statistical methods will be selected for each research question depending upon the nature of the data distribution as well as the effect that is to be characterized. Categorical variables will be presented as absolute numbers and percentages, while continuous variables will be described by number of observations, mean, standard deviation, median and range (minimum and maximum). While maximum effort will be put into the minimization of missing data, the remote nature of the study and the reliance on self-reporting by the subjects might lead to the presence of missing data. Where necessary, missing data mechanisms will be investigated and imputation methods will be used or robust statistical methods such as bootstrap estimation employed. The count and percentage of known missing data will be described. Analyses and results between and within the study conditions will also be reported overall and separately for the different strata (e.g., normal-risk vs. high-risk population). Additionally, with the exception of the baseline data, all data will be analysed and reported within each period of the trial with pooling of the data only in specifically mentioned cases in the sections below. All statistical confidence intervals will be two-sided and at the 95% level. P-values, or similar measures resulting from bootstrap methods, will be considered significant if less than 0.05 with two-sided testing and if less than 0.025 with one-sided testing. No adjustments for multiplicity of tests are planned. For both the primary and secondary endpoints (where relevant), we will investigate the potential effect of COVID-19 vaccination among some subjects on the analyses of interest using sensitivity analyses. This may include truncation of certain data occurring after vaccination events as part of these sensitivity analyses. Further details will be captured in the statistical analysis plan.

*Development and validation of algorithms*

As indicated in section 3.3, the algorithms applied in both the control and experimental condition will continually be developed during the trial with specific versions being released and evaluated throughout. During the Learning Phase and Period 1, first versions of the algorithms will be implemented which have been developed using data collected from previous studies and existing users of the Ava bracelet. Data collected during the Learning Phase will then be used to develop second versions of the algorithms which will be implemented and statistically evaluated in Period 2. When all subject follow-up is complete and all data is collected, final versions of the algorithms will be developed. As such, the Learning Phase will primarily be used to collect both physiological and symptom data to train both algorithms, Period 1 to gain further understanding of the first version of the algorithm and enable blinded sequences, and Period 2 will be used to statistically evaluate the second version of the algorithm as specified in the primary objective.

For these analyses, the duration of implementation of different versions of the algorithms will be taken into account. As such, the performance of the implemented algorithms will be calculated using data collected during its implementation. In addition, the performance of different versions of the algorithms can be assessed by applying them retrospectively to all of the collected raw data. In this way, the algorithms will be judged as actually implemented in the trial (*de facto* estimation) and under a hypothetical condition wherein the final developed algorithm is treated as having been implemented for each subject (*de jure* estimation).

*Comparison between study conditions and within-subjects*

For the primary comparison between study conditions, the control condition will act as a reference group towards the experimental condition for Period 2. By directly comparing both study conditions within the crossover design, the added benefit can be assessed of including the Ava bracelet data for the detection of potential SARS-CoV-2 infections. In addition to the evaluation within Period 2, control vs. experimental comparisons will be made using both periods via application of bootstrapping methods detailed further below. In addition, a within-subjects analysis will be performed using all subjects as they will all wear and synchronise the bracelet throughout the trial. This allows for an extra within-subject analysis of the ability of the algorithm based on the symptom data with or without the bracelet data to detect early and asymptomatic SARS-CoV-2 infection.

*Analysis sets*

Several analysis sets will be defined which will be used for the analyses described in the following sections:

- **Intention-to-treat set**: All subjects randomized to one of the two study sequences. For the analysis, subjects will be considered part of the study sequence that they were originally randomized to. Note that when a subject has their first SARS-CoV-2 infection (as confirmed through a positive PCR/antigen or serology test), they will no longer be part of the analysis going forward because they have met the primary outcome.
- **Efficacy analysis set**: All compliant subjects randomized to one of the two study sequences, where compliance is defined as having entered symptom data in the Ava COVID-RED app on at least 80% of the days of Period 2 **and**, when in the experimental condition, having worn and synchronised the Ava bracelet on at least 80% of the days of Period 2.
- **Partial compliance set**: All subjects randomized to one of the two study sequences that were partially compliant; which is defined as having entered symptom data in the Ava COVID-RED app on at least 80% of the days of Period 2 **or**, when in the experimental condition, having worn and synchronised the Ava bracelet on at least 80% of the days of Period 2.
- **Safety analysis set:** All subjects who were randomized and received an Ava bracelet.

## Baseline Summary

Descriptive analyses of baseline characteristics detailed in section 8.1.3 will be carried out on the Intention-to-treat, Efficacy analysis and Partial compliance sets. Where deemed relevant, analyses will also be executed within selected subsets of these analysis sets. Specific subsets will be listed within the Statistical Analysis Plan. Results will be presented in tables where possible. Where percentages are to be calculated, the number of subjects in the condition within the specified analysis (sub)set will be used as the denominator. To assess whether differences between groups are statistically significant, paired or unpaired (depending on between or within group comparisons) two-sample t-tests and chi-squared tests will be used where appropriate.

## Primary Analysis of Primary Endpoint(s)

The primary analysis of the primary endpoint will compare the performance of the algorithm applied in each study condition to detect if subjects were ever infected during Period 2. This analysis will be performed on both the Intention-to-treat and Efficacy analysis sets, within each period and stratified per risk group. For the subjects within each study condition, the daily prediction of possible SARS-CoV-2 infection and the results of PCR/antigen and serology tests will be summarized over the whole period per subject to determine (1) if a subject was ever judged to have a high risk for a potential SARS-Cov-2 infection (i.e. received a red-light indicator from the Ava COVID-RED app), and (2) if a subject was ever confirmed to have a SARS-CoV-2 infection by PCR/antigen or serology testing. For this comparison, parameters such as sensitivity, specificity, positive predictive value, and negative predictive value will be calculated.

To confirm whether a subject was ever infected in Period 2 with SARS-CoV-2, the serology status at the end of Period 2 for each subject still alive at that time will be used as the gold standard. Those with a previous positive test result will be excluded from the analysis. For subjects not alive at the end of Period 2, the status of available SARS-CoV-2 virus tests during Period 2 will be used (positive at any time) and for those without any test results, a physician diagnosis during Period 2 will be accepted provided there was a supportive lung CT scan used. All other subjects will be summarized for transparency but excluded from the estimation for sensitivity, specificity, positive predictive value and negative predictive value. The following quantities are needed for these statistics:

- **Predicted condition positive:** The total number of subjects that obtained a red-light indicator from the Ava COVID-RED app at least once during Period 2.
- **Predicted condition negative:** The total number of subjects that never obtained a red-light indicator from the Ava COVID-RED app during Period 2.
- **Condition positive:** The total number of subjects who tested positive at least once using SARS-CoV-2 virus testing during Period 2 OR who had a positive serology test at the end of Period 2.
- **Condition negative:** The total number of subjects who never tested positive for the SARS-CoV-2 virus during Period 2 AND who had a negative serology test at the end of Period 2.

To compare statistics such as the sensitivity and specificity between the two study conditions while taking into account the two strata, a Cochran-Mantel-Haenszel test will be used. A within-person (*de jure*) comparison will also be made by retrospectively evaluating the performance of the fully developed algorithm with and without the bracelet data included for all subjects. In this case, the sensitivity and specificity statistics will be compared using a McNemar’s test. Statistical analyses will also be performed on data from both Periods 1 and 2 combined. See Section 10.4 for details on this analysis.

## Secondary Analysis of Primary Endpoint(s)

The secondary analysis of the primary endpoint will compare which of the two algorithms between study conditions has superior performance characteristics for detecting early or asymptomatic SARS-CoV-2 indication or infection. Time-to-event analyses will be performed using the Intention-to-treat, Efficacy analysis and Partial compliance set. Analyses will be performed within each study period and stratified per risk group. Kaplan-Meier plots will be constructed for visual support. An event will be defined as either the first indication (red-light indicator from the Ava COVID-RED app) or first infection (as confirmed by PCR/antigen testing) of SARS-CoV-2 of a subject during the study period. Subjects lost to follow-up or reaching the end of the period without infection will be censored. Stratified log-rank tests will be performed within each period to assess whether the experimental condition shows a statistically significant improvement (or lack thereof) in time to indication or infection as compared to the control condition with a median difference used to state the effect size. If the median is not yet reached in either study condition then a different quantile will be used. If other variables are to be taken into account, Cox proportional hazards models will be used where assumptions are met.

This same analysis will also be performed retrospectively in all subjects where the time to first indication or infection will be assessed using the algorithm based on only the symptoms data or both the symptoms and bracelet data within each subject. Because the measurement methods are correlated (both physically and in data collection), a within-person comparison can be made using paired statistical methods. If assumptions are satisfied, a Wilcoxon signed rank test will be used for this analysis; otherwise, other paired tests will be considered.

## Synthesis of evidence

To benefit from the crossover design and synthesize the results of a chosen test statistic over both study periods, it has to be taken into account that there is a missing-data mechanism by design. Subjects with SARS-CoV-2 infection in the Learning Phase will have their data excluded from Period 1 and Period 2. Likewise, Subjects with SARS-CoV-2 infection in Period 1 will be have their data excluded from Period 2. As such, traditional crossover analysis methods and adjustments for missing data are not sufficient and bootstrap methods will be used. To indicate how the bootstrap approach can be used it is illustrated for the ever-infected analysis mentioned in section 10.2. Table 6 shows example data for the ever-infected analysis from which **predicted condition positive**, **predicted condition negative**, **condition positive**, and **condition negative** can be derived.

Table 6: Example data for ever infected analysis

| UID | Condition  Period 1 | Observed outcome P1 | True outcome P1 (serology) | Condition  Period 2 | Observed outcome P2 | True outcome P2 (serology) |
| --- | --- | --- | --- | --- | --- | --- |
| 1 | 1 | 0 | 0 | 0 | 0 | 1 |
| 2 | 0 | 1 | 1 | NA | NA | NA |
| 3 | 1 | 0 | 1 | NA | NA | NA |
| … |  |  |  |  |  |  |
| 20,000 | 0 | 0 | 0 | 1 | 1 | 1 |

### Bootstrap approach

Using data from Table 6, sample statistics, denoted by $s_{ij}$, can be calculated, where $i=1$ denotes the experimental condition, $i=2$ the control condition, $j=1$ study Period 1 and $j=2$ study Period 2 (see Table 7). The sample statistics in question could be sensitivity or specificity for instance. In a normal statistical analysis, distributional assumptions are used to provide uncertainty about the sample statistics obtained in the study. A bootstrapping approach does not require these distributional assumptions.

Bootstrapping is a resampling method in which sample statistics are calculated multiple times using new samples drawn with replacement from the original dataset. This leads to an empirical distribution of the sample statistics which can be used to quantify the uncertainty. In the current study, paired observations are drawn (i.e. both the outcome of Period 1 and Period 2 are jointly sampled for each subject).

Table 7: Notation of statistics per arm and period

|  | Period 1 | Period 2 |
| --- | --- | --- |
| Sequence 1 | Experimental Treatment | Control Treatment |
| Sequence 2 | Control Treatment | Experimental Treatment |

|  | Period 1 | Period 2 |
| --- | --- | --- |
| Experimental Condition | $s_{11}$ | $s_{12}$ |
| Control Condition | $s_{21}$ | $s_{22}$ |

### Weighting of evidence

To synthesize the sample statistics and treatment effect over both periods while taking into account the missing data structure, a weighting procedure can be used. The weights associated with each study condition and period combination are denoted by $w_{ij}$. The weights are equal to the number of observations used to calculate the corresponding test statistic. For example, if there are no dropout in Periods 1 and 2 but we do find respectively 500 and 600 positive serology tests at the end of Period 1 for the experimental condition and the control condition, then $w_{\cdot1}$ is 10.000 and $w_{\cdot2}$ is 8900. Because 500 people in Period 1 in the experimental condition had a positive serology test, the sample size for the control condition in Period 2 is reduced by 500. Aggregated statistics for the study conditions can be calculated by $\bar{S}_{1}+\bar{S}_{2}$.

$$\bar{S}_{1}=\frac{{w_{\cdot1}*(S}_{11}-S_{21})}{w_{\cdot1}+w_{\cdot2}}$$

$$\bar{S}_{2}=\frac{{w_{\cdot2}*(S}_{12}-S_{22})}{w_{\cdot1}+w_{\cdot2}}$$

For every bootstrap sample, $\bar{S}_{1}$ and $\bar{S}_{2}$ can be computed and a variance estimate for $\bar{S}_{1}+\bar{S}_{2}$ can be obtained from this empirical distribution. Note that the weights also vary for each bootstrap sample depending upon the number of Period 1 infections that were sampled.

## Analysis of Secondary Endpoint(s)

Secondary study endpoints mentioned in section 8.1.2 will be analysed and compared within the Intention-to-treat, Efficacy analysis and Partial compliance sets within each period. Where deemed relevant, analyses will also be executed within selected subsets of these analysis sets. Specific subsets will be listed within the Statistical Analysis Plan. Results will be presented in tables where possible. Where percentages are to be calculated, the number of subjects in the condition within the specified analysis (sub)set will be used as the denominator.

Where assumptions are met, Cochran-Mantel-Haenszel tests stratified by the risk strata will be performed in each period for multiple secondary endpoints, including:

- The number and percentage of subjects that obtained a red-light indicator by the applied algorithm at least once.
- The number and percentage of subjects with at least one positive SARS-CoV-2 virus test result during follow-up.
- The number and percentage of subjects that donated a blood sample for a SARS-CoV-2 antibody test at baseline, after the Learning Phase and after both periods.
- The number and percentage of subjects with a positive SARS-CoV-2 antibody test at baseline, after the Learning Phase and after both periods.
- The number and percentage of subjects with at least one positive SARS-CoV-2 virus test result during each period OR a positive SARS-CoV-2 antibody test result within those periods.
- The number and percentage of subjects without a positive SARS-CoV-2 virus test result during each period AND a negative SARS-CoV-2 antibody test result within those periods.
- The number and percentage of subjects that have reported experiencing each of the symptoms listed in the Ava COVID-RED app at least for one day during the study.
- The number and percentage of subjects that have reported experiencing each of the symptoms corresponding by the RIVM guidelines at least for one day during the study.
- The number of subjects who reported a positive SARS-CoV-2 virus test during the study and the percentage of subjects who reported a positive SARS-CoV-2 virus test during the study, with the number of subjects ever receiving a PCR test during the study as the denominator.

Where assumptions are met, t-tests will be performed in each period for multiple secondary endpoints, including:

- The average and median number of times a single subject obtained a red-light indicator by the applied algorithm.
- The average and median number of continuous days that each symptom in the Ava COVID-RED app was reported to be experienced.
- The average and median number of continuous days that each symptom corresponding to the RIVM guidelines was reported to be experienced.
- The average (and variance) number of days between a red-light indicator of the applied algorithm and the SARS-CoV-2 virus test result, stratified for tests done via the GGD and central laboratory, and also stratified for testing methodology.
- The average (and variance) number of days between a red-light indicator of the applied algorithm and the taking of a SARS-CoV-2 virus test, stratified for tests done via the GGD and central laboratory, and also stratified for testing methodology.
- Number of total SARS-CoV-2 virus tests taken by subjects, stratified for tests taken by GGD and/or central laboratory, and also stratified by testing methodology if applicable (PCR vs. antigen tests).
- The number of total positive SARS-CoV-2 virus tests and percentage of positive tests, with the number of total tests within the study taken as a denominator. This will also be stratified for the different testing methodologies if applicable.
- Average and median duration of Ava bracelet and/or Ava COVID-RED app use (number of days between first day of use and last day of use)
- Compliance rate: percentage of days (as compared to duration of use) that Ava bracelet data was synced to Ava COVID-RED app or symptoms were entered. Upon inspection of the data, other modes (like Poisson regression) will also be considered.

For other relevant endpoints (e.g. survival status of subjects at end of follow-up) appropriate statistical methods (e.g. survival analysis) will be used. Further details will be indicated within a separate statistical analysis plan.

## Analysis of Safety

As the Ava bracelet is well-characterized within its marketed indication, we expect very few reports of ADEs and for those to be minor (e.g., skin rash after wearing the Ava bracelet). ADEs will be listed and no statistical summary of these data are planned.

## Interim analysis

The applied algorithms in both study conditions will be updated using data gathered throughout the study and a new version will be implemented in Period 2 of the study. When evaluating the different versions of the algorithm as mentioned above, we will essentially obtain interim *de facto* estimates of the performance of these algorithms. The final version of the algorithm will also be retrospectively applied on the data of the subjects within the considered analysis sets to obtain a final *de jure* estimate of the performance. As this is not a traditional hypothesis testing study, there will not be any adjustments to the Type 1 error level (alpha) to account for any interim statistical analysis of trial data.

# ETHICAL CONSIDERATIONS

## Regulation statement

The study will be conducted in full conformance with the principles of the “Declaration of Helsinki” (64th WMA General Assembly, Fortaleza, Brazil, October 2013) or with the laws and regulations of the country in which the research is conducted, whichever affords the greater protection to the individual. The study will comply with the General Data Protection Regulation EU 2016/679 (GDPR); in Dutch: Algemene Verordening Gegevensbescherming (AVG) and the Dutch Act on Medical Devices (Besluit Medisch Hulpmiddelen).

## Recruitment and consent

Recruitment will be completely remote and take place via post, email and electronic web portals. In this way, risk of SARS-CoV-2 infection is minimized as much as possible for those wanting to participate in the trial. Subjects will initially be approached from previously studied cohorts from the Sponsor-Investigator as well as via public campaigns. Members of existing cohorts will be contacted through email or mail to inform them about the COVID-RED study. In the letter or e-mail, they will be invited to visit the COVID-RED web portal if they would be interested to participate in the study. For the public campaign, an online URL link will be available for any individual who wants to know more about the study and would like to participate. In the COVID-RED web portal, a survey will be completed in which it will be verified if the subject meets the criteria to join the study. Subjects who do not meet the eligibility criteria or if they meet the criteria within a strata that is filled will be informed that they are not able to join the study in real-time via the COVID-RED web portal.

When the subject is eligible, they will be provided with further information on participation in the study. Each subject will be informed that their participation in the study is voluntary, that he/she may withdraw from the study at any time and that withdrawal of consent will not have any impact.. The subject information sheet and consent form (ICF) describe the study and provide sufficient information for the subject to make an informed decision about their participation in the study. The subject will be offered the possibility to get in contact with study team staff to discuss/answer any questions and/or concerns regarding participation in the study. Only if the subject voluntarily agrees to sign the ICF and has done so in the COVID-RED web portal, then he/she may enter the study. Subjects who are unable to read Dutch are not able to join the study. The information sheet and consent form that will be used must be approved by the reviewing EC.

## Objection by minors or incapacitated subjects (if applicable)

Not applicable

## Benefits and risks assessment, group relatedness

The risks associated with participation can be considered minimal. Subjects will be treated with the standard of care and will receive the study intervention (Ava COVID-RED app and the Ava bracelet) on top of their standard care. Please refer to section 12 for the structured risk analyses.

## Compensation for injury

The sponsor/investigator has a liability insurance which is in accordance with the legal requirements in the Netherlands

## Incentives (if applicable)

Subjects are not reimbursed for their participation. During their participation, subjects have full access to all medical care as they would have if they were not a subject in a study.

# ADMINISTRATIVE ASPECTS, MONITORING AND PUBLICATION

## Handling and storage of data and documents

The collection and processing of personal data from subjects enrolled in the study will be limited to those data that are necessary to fulfil the objectives of the study. The data is collected and processed with adequate precautions to ensure confidentiality and compliance with applicable data privacy protection laws and regulations. Appropriate procedures are implemented to protect the personal data against unauthorized disclosure, access, loss or alteration.

Clinical data will be captured using an eCRF, developed with Electronic Data Capture technology (EDC), the COVID-RED web portal, the Ava COVID-RED app and the Ava bracelet and laboratory results (PCR/antigen and serology antibody testing). The eCRF and COVID RED web portal are both hosted in Castor EDC (APPENDIX 2).

Essential study documents will be stored on the server at Julius Clinical (JC storage) and this location is also used to store any data exports or reports. Personal data stored on the server of Julius Clinical will be separated from pseudonymized data and will only be accessible to the Data manager, Helpdesk of Julius Clinical, and the Julius Clinical Data Transfer Lead and his/her delegate (APPENDIX 2). The Data Transfer Lead (who is an employee within Julius Clinical as well as one back-up) is responsible for the day-to-day management of communications to study participants and for data transfers between Julius Clinical, the central lab and the fulfilment partner (Mailstreet).Julius Clinical will keep a log of which persons are authorized to have access to personal data. Persons will have access only to what is necessary to perform their study specific duties.

Basic socio-demographic data on screening failures will be collected with permission of the screening persons for statistically characterizing the entire screened population. These data will lack any personal identifiers.

Data from the eCRF and COVID-RED web portal will be pseudonymized (subjects will be assigned a unique identification number in Castor EDC) and stored in a study database (Castor EDC). Only authorised study staff (Sponsor/Investigator and Julius Clinical) will be allowed to enter data into the eCRF and make changes to eCRF data. In addition, only the Data manager and Physicians from UMCU carrying out the interviews will have access to subjects’ personal data (Sponsor/Investigator and Julius Clinical) (APPENDIX 2).

The key to connect the identification number to the subject will be stored in a separate section of the EDC and will only be available for a limited number of study staff from the Sponsor/Investigator and Julius Clinical, such as the Data manager, Helpdesk of Julius Clinical, the Julius Clinical Data Transfer Lead and his/her delegate and Physicians at UMCU carrying out the interviews in case of lost-to-follow up, hospitalization, or adverse device effect scenarios (APPENDIX 2).

Subject personal data (name and address) will also be shared with the vendor (Mailstreet) responsible for sending the onboarding and study supplies to the subjects. Since recruitment and follow-up will be completely remote, supplies will need to be sent to the subjects’ home addresses (APPENDIX 2).

The Julius Clinical Data Transfer Lead and his/her delegate will request additional personal data (required for reporting to the GGD) in case the PCR sample taken by the subject themself is positive. SARS-CoV-2 is a notifiable disease in the Netherlands and therefore all subjects who tested positive must be reported to the GGD, so that the GGD can initiate contact-tracing (see section 3.5 and 8.4.12).

Data points that originate from a third party database (e.g., Ava COVID-RED app, Ava bracelet and laboratory results from SARS-CoV-2 serology antibody testing), will be transferred via secure means to the investigator. Transferred data will not contain personal identifiers as only trial-specific identifiers will be used to link data between parties.

Data points that are not considered part of the eCRF (e.g., derived data points and administrative data points) will be automatically calculated or entered by authorised staff of Sponsor, Investigator or it’s designee.

All changes made to the eCRF data will be captured via an electronic audit trail, indicating at least date and time of change, the reason for changing the data, the individual that made the change and the old and new data value.

Source data is not applicable for this trial as all data will either be directly entered into the COVID-RED web portal, eCRF, Ava COVID-RED app for the purposes of this study or be laboratory test results.

In compliance with relevant guidelines, the study team will maintain all eCRFs as well as all study documents as specified by the applicable regulatory requirement(s). These records must be readily available for audit or inspection.

If it becomes necessary for the appropriate regulatory authority to review any documentation relating to this study, the study team must permit access to such reports.

All essential study documents and data will be retained for 20 years after completion of the study.

## Monitoring and Quality Assurance

No monitoring visits will be conducted for the study. The Investigator and his study team are responsible to ensure that the study is conducted in accordance with the protocol and local regulations. The eCRF data and Ava COVID-RED app data will be reviewed internally by Clinical Team members, Data Management, Statistics, Medical and Scientific staff or their designee and, if necessary, appropriate actions will be implemented in case data entry issues are detected. Once data are concluded to be complete and accurate, the eCRF data will be locked, meaning that the data will become read-only. COVID-RED web portal data, eCRF data, aggregated Ava bracelet data, Ava COVID-RED app data and laboratory data will only be accessible and verifiable by the authorized study team members and adequate back-up and security measures are implemented to prevent loss of data or unauthorised access to the data.

## Amendments

Amendments are changes made to the research after a favourable opinion by the accredited EC has been given. All amendments will be notified to the EC that gave a favourable opinion.

## Annual progress report

Not applicable.

## Temporary halt and (premature) end of study report

The investigator/sponsor will notify the accredited EC of the end of the study within a period of 8 weeks. The end of the study is defined as receipt of the last patient’s last sample (fingerprick blood for SARS-CoV-2 serology (antibody) testing).
The sponsor will notify the EC immediately of a temporary halt of the study, including the reason for such an action.

In case the study is ended prematurely, the sponsor will notify the accredited EC within 15 days, including the reason(s) for the premature termination.

## Public disclosure and publication policy

This study will be registered on the clincialtrials.gov and/or clincialtrials.eu websites, which are registries of studies conducted in Europe, the United States and around the world.

The results of the study will be reported in a Study Report generated by the Sponsor-Investigator or Designee. The study results will be publicly disclosed and published independent of the outcome of the study in scientific, peer-reviewed, international journals and at international conferences.

# STRUCTURED RISK ANALYSIS

## Potential issues of concern

The structured risk analysis is kept short, since in this interventional study no physiological or medicinal effects are accomplished. The use of the Ava bracelet and the Ava COVID-RED app are intended to monitor general health and wellness of the subjects. More information on risk analyses is available in the most recent version of the Ava COVID IMDD.

a. Level of knowledge about mechanism of action

The Ava bracelet is a wearable device with three sensors that sits on the dorsal side of the user’s wrist. The bracelet measures and records physiological parameters including heart rate, breathing rate, heart rate variability, wrist skin temperature, perfusion, and sleep quality (36–38).

b. Previous exposure of human beings with the test product(s) and/or products with a similar biological mechanism

The device relies on the same hardware (Hardware Bracelet Generation v2.0) as the commercially marketed Ava Fertility Tracker.

Some subjects wearing the Ava Fertility Tracker snugly for extended time have reported skin irritation and/or sensitization due to rubbing and friction; thus, the User Manual for the current hardware indicates that people with cholinergic urticaria (sweat induced allergies) cannot use the device.

c. Can the primary or secondary mechanism be induced in animals and/or in *ex-vivo* human cell material?

Not applicable; no pre-clinical studies have been performed with the Ava bracelet for the detection of COVID-19 infection.

d. Selectivity of the mechanism to target tissue in animals and/or human beings

Not applicable; no effect on human tissue is expected. The device is worn externally and is aimed at monitoring general health and well-being.

e. Analysis of potential effect

No medicinal effect is expected. The device is aimed at monitoring users’ general health and wellbeing.

f. Pharmacokinetic considerations

Not applicable.

g. Study population

The intended patient population are adults of at least 18 years old who want to monitor their general health and wellness. Subjects suffering from cholinergic urticaria will be excluded from the study as the current User Manual for the Ava bracelet excludes these individuals from using the device. Pregnant women are also excluded, as the algorithm for detecting symptoms in line with a potential COVID-19 infection is not tuned for the physiological changes that occur over the course of pregnancy. Individuals with tendonitis, carpal tunnel syndrome, or other musculoskeletal disorders should consult with their doctor prior to enrolling in the study and using the Ava bracelet.

h. Interaction with other products

The Ava bracelet must be kept away from high frequency devices as this may interfere with operation. Subjects with electronic implanted devices such as a pacemaker are excluded from the study.

i. Predictability of effect

The Ava bracelet (i.e., the hardware) operates identically to the CE-marked device, the Ava Fertility Tracker. After data from the device is synchronised with the backend servers each day, a machine learning algorithm analyses the user’s recent data and classifies the user’s health. In the CE-marked Ava Fertility Tracker, the underlying algorithm classifies the user’s fertility status for a given day as fertile or infertile; prior research has demonstrated the algorithm can detect the user’s fertile window with 90% accuracy (95% Confidence Interval 0.89 to 0.92)(37). For this clinical trial, the same Ava Hardware Bracelet Generation 2.0 will be used to generate a COVID-19-related indication. A novel algorithm, based on proprietary data gathered by real-world users of the Ava Fertility Tracker, will be deployed to detect significant variation in physiological parameters, which could signal a potential COVID-19 infection. This indicator is shown in-app to the user in real-time during the Learning Phase and when they are in the experimental condition.

j. Can effects be managed?

Antidotes or antagonist are not applicable for this intervention. For the follow up on potential COVID-19 infection as flagged by the Ava COVID-RED app, subjects are instructed by study staff and onboarding instructions to utilize standard medical care.

## Synthesis

The risks for the intervention (wearing the Ava bracelet and interaction with the Ava COVID-RED app) are expected to be very minimal. Subjects will be treated with the standard of care and will receive the study intervention on top of their standard care.

Subjects will be instructed to discontinue wearing the Ava bracelet and contact the study team in case they experience any signs of allergic reaction at their wrist, feel soreness, tingling, numbness, burning or stiffness in their hands or wrists while or after wearing the bracelet.

Overall, the burden for the subjects is assessed as small and is justified given the importance of retrieving more information on a potential method in early detection of COVID-19. The expected benefit is large, as the algorithms trained on the obtained data recordings from the Ava bracelet are expected to recognize COVID-19 earlier than clinical symptoms. The latter would allow for earlier isolation and stratification as well as monitoring of COVID-19 affected patients preventing further spread and allowing for appropriate healthcare.

# REFERENCES

1. Kretzschmar ME, Rozhnova G, Bootsma MCJ, van Boven M, van de Wijgert JHHM, Bonten MJM. Impact of delays on effectiveness of contact tracing strategies for COVID-19: a modelling study. Lancet Public Heal. 2020 Aug;5(8):e452–9.

2. Wiersinga WJ, Rhodes A, Cheng AC, Peacock SJ, Prescott HC. Pathophysiology, Transmission, Diagnosis, and Treatment of Coronavirus Disease 2019 (COVID-19): A Review. JAMA. 2020 Aug;324(8):782–93.

3. Johansson M, Quandelacy T, Kada S. Controlling COVID-19 requires preventing SARS-CoV-2 transmission from people without symptoms. Submitted. 2020;

4. Moghadas SM, Fitzpatrick MC, Sah P, Pandey A, Shoukat A, Singer BH, et al. The implications of silent transmission for the control of COVID-19 outbreaks. Proc Natl Acad Sci U S A. 2020 Jul;117(30):17513–5.

5. WHO. Report of the WHO-China Joint Mission on Coronavirus Disease 2019 (COVID-19). [Internet]. 2020. Available from: https://www.who.int/publications-detail/report-of-the-who-china-joint-mission-on-coronavirus-disease-2019-(covid-19)

6. Zhang J-J, Dong X, Cao Y-Y, Yuan Y-D, Yang Y-B, Yan Y-Q, et al. Clinical characteristics of 140 patients infected with SARS-CoV-2 in Wuhan, China. Allergy. 2020 Jul;75(7):1730–41.

7. Guan W-J, Ni Z-Y, Hu Y, Liang W-H, Ou C-Q, He J-X, et al. Clinical Characteristics of Coronavirus Disease 2019 in China. N Engl J Med. 2020 Apr;382(18):1708–20.

8. Sahu K, Mishra A, Lal A. Comprehensive update on current outbreak of novel coronavirus infection (2019-nCoV) [Internet]. Ann Transl Med. 2020. Available from: http://atm.amegroups.com/article/view/36814

9. Guan W, Ni Z, Hu Y, Liang W, Ou C, He J, et al. Clinical characteristics of 2019 novel coronavirus infection in China. medRxiv [Internet]. 2020 Jan 1;2020.02.06.20020974. Available from: http://medrxiv.org/content/early/2020/02/09/2020.02.06.20020974.abstract

10. Chen G, Xie J, Dai G, Zheng P, Hu X, Lu H, et al. Validity of Wrist and Forehead Temperature in Temperature Screening in the General Population During the Outbreak of 2019 Novel Coronavirus: a prospective real-world study. medRxiv [Internet]. 2020 Jan 1;2020.03.02.20030148. Available from: http://medrxiv.org/content/early/2020/03/06/2020.03.02.20030148.abstract

11. Karjalainen J, Viitasalo M. Fever and cardiac rhythm. Arch Intern Med. 1986 Jun;146(6):1169–71.

12. Downey CL, Chapman S, Randell R, Brown JM, Jayne DG. The impact of continuous versus intermittent vital signs monitoring in hospitals: A systematic review and narrative synthesis. Int J Nurs Stud. 2018 Aug;84:19–27.

13. Haahr-Raunkjær C, Meyhoff CS, Sørensen HBD, Olsen RM, Aasvang EK. Technological aided assessment of the acutely ill patient - The case of postoperative complications. Eur J Intern Med. 2017 Nov;45:41–5.

14. Olsen RM, Aasvang EK, Meyhoff CS, Dissing Sorensen HB. Towards an automated multimodal clinical decision support system at the post anesthesia care unit. Comput Biol Med. 2018 Oct;101:15–21.

15. Weenk M, van Goor H, Frietman B, Engelen LJ, van Laarhoven CJ, Smit J, et al. Continuous Monitoring of Vital Signs Using Wearable Devices on the General Ward: Pilot Study. JMIR mHealth uHealth. 2017 Jul;5(7):e91.

16. Tomasic I, Tomasic N, Trobec R, Krpan M, Kelava T. Continuous remote monitoring of COPD patients-justification and explanation of the requirements and a survey of the available technologies. Med Biol Eng Comput. 2018 Apr;56(4):547–69.

17. Abdel Latif Al-Shwaheen T, Hau Y. A new model for tracking and detection of deterioration of vital signs based on artificial neural network. J Theor Appl Inf Technol. 2019;97(14).

18. Clifton L, Clifton DA, Pimentel MAF, Watkinson PJ, Tarassenko L. Predictive monitoring of mobile patients by combining clinical observations with data from wearable sensors. IEEE J Biomed Heal informatics. 2014 May;18(3):722–30.

19. Steinhubl SR, Feye D, Levine AC, Conkright C, Wegerich SW, Conkright G. Validation of a portable, deployable system for continuous vital sign monitoring using a multiparametric wearable sensor and personalised analytics in an Ebola treatment centre. BMJ Glob Heal. 2016;1(1).

20. Loftus TJ, Tighe PJ, Filiberto AC, Balch J, Upchurch GRJ, Rashidi P, et al. Opportunities for machine learning to improve surgical ward safety. Am J Surg. 2020 Oct;220(4):905–13.

21. Vistisen ST, Johnson AEW, Scheeren TWL. Predicting vital sign deterioration with artificial intelligence or machine learning. Vol. 33, Journal of clinical monitoring and computing. Netherlands; 2019. p. 949–51.

22. Shen J, Zhang CJP, Jiang B, Chen J, Song J, Liu Z, et al. Artificial Intelligence Versus Clinicians in Disease Diagnosis: Systematic Review. JMIR Med informatics. 2019 Aug;7(3):e10010.

23. Alloghani M, Al-Jumeily D, Aljaaf AJ, Khalaf M, Mustafina J, Tan SY. The Application of Artificial Intelligence Technology in Healthcare: A Systematic Review. In: International Conference on Applied Computing to Support Industry: Innovation and Technology. Springer; 2019. p. 248–61.

24. Bol N, Helberger N, Weert JCM. Differences in mobile health app use: A source of new digital inequalities? Inf Soc. 2018;34(3):183–93.

25. Jeffrey B, Bagala M, Creighton A, Leavey T, Nicholls S, Wood C, et al. Mobile phone applications and their use in the self-management of Type 2 Diabetes Mellitus: a qualitative study among app users and non-app users. Diabetol Metab Syndr. 2019;11:84.

26. Hollander JE, Carr BG. Virtually perfect? Telemedicine for COVID-19. N Engl J Med. 2020;382(18):1679–81.

27. Smith AC, Thomas E, Snoswell CL, Haydon H, Mehrotra A, Clemensen J, et al. Telehealth for global emergencies: Implications for coronavirus disease 2019 (COVID-19). J Telemed Telecare. 2020;1357633X20916567.

28. Greenhalgh T, Wherton J, Shaw S, Morrison C. Video consultations for covid-19. BMJ [Internet]. 2020;368. Available from: https://www.bmj.com/content/368/bmj.m998

29. Allen WE, Altae-Tran H, Briggs J, Jin X, McGee G, Shi A, et al. Population-scale longitudinal mapping of COVID-19 symptoms, behaviour and testing. Nat Hum Behav. 2020 Sep;4(9):972–82.

30. Nguyen LH, Drew DA, Graham MS, Joshi AD, Guo C-G, Ma W, et al. Risk of COVID-19 among front-line health-care workers and the general community: a prospective cohort study. Lancet Public Heal. 2020 Sep;5(9):e475–83.

31. Timmers T, Janssen L, Stohr J, Murk JL, Berrevoets MAH. Using eHealth to Support COVID-19 Education, Self-Assessment, and Symptom Monitoring in the Netherlands: Observational Study. JMIR mHealth uHealth. 2020 Jun;8(6):e19822.

32. Soriano JB, Fernández E, de Astorza Á, Pérez de Llano LA, Fernández-Villar A, Carnicer-Pont D, et al. Hospital Epidemics Tracker (HEpiTracker): Description and pilot study of a mobile app to track COVID-19 in hospital workers. JMIR public Heal Surveill. 2020 Sep;6(3):e21653.

33. Zhang H, Dimitrov D, Simpson L, Plaks N, Singh B, Penney S, et al. A Web-Based, Mobile-Responsive Application to Screen Health Care Workers for COVID-19 Symptoms: Rapid Design, Deployment, and Usage. JMIR Form Res. 2020 Oct;4(10):e19533.

34. Metcalf D, Milliard STJ, Gomez M, Schwartz M. Wearables and the Internet of Things for Health: Wearable, Interconnected Devices Promise More Efficient and Comprehensive Health Care. IEEE Pulse. 2016;7(5):35–9.

35. Seshadri DR, Davies E V, Harlow ER, Hsu JJ, Knighton SC, Walker TA, et al. Wearable sensors for COVID-19: a call to action to harness our digital infrastructure for remote patient monitoring and virtual assessments. Front Digit Heal. 2020;2:8.

36. Shilaih M, Goodale BM, Falco L, Kübler F, De Clerck V, Leeners B. Modern fertility awareness methods: wrist wearables capture the changes in temperature associated with the menstrual cycle. Biosci Rep [Internet]. 2018 Nov 30;38(6). Available from: https://doi.org/10.1042/BSR20171279

37. Goodale BM, Shilaih M, Falco L, Dammeier F, Hamvas G, Leeners B. Wearable sensors reveal menses-driven changes in physiology and enable prediction of the fertile window: an observational study. J Med Internet Res. 2019;21(4):e13404.

38. Shilaih M, de Clerck V, Falco L, Kübler F, Leeners B. Pulse rate measurement during sleep using wearable sensors, and its correlation with the menstrual cycle phases, a prospective observational study. Sci Rep. 2017;7(1):1–7.

# VERSION HISTORY

| Protocol version and date | Summary of changes | Rationale for change |
| --- | --- | --- |
| Version 1.0 date 14Dec2020 | NA, initial version | NA, initial version |
| Version 1.1 date 08Jan2021 | Update procedures and an endpoint for subjects that will get vaccinated for COVID 19 when they are in the study.  Additional exclusion criterion add that subjects who are already vaccinated prior to entry the study cannot join the study  Update section 9 on the reporting of adverse events to the EC and Competent Authority.  Update of section 11.5 since for this non-WMO study only the liability insurance will be applicable.  Update section 12 to better explain data collection, storage and who has access to the data. | Updates upon feedback of the EC |
| Version 1.2 date 22Jan2021 | Name of the central lab added.  Update of section 12.1 to better explain who has access to personal identifiable data. | Updates upon feedback of the EC |
|  |  |  |

# APPENDIX 1

| Observation point | Recruitment | Enrollment | Delivery of Study Materials | Study Period | | | End of Study |
| --- | --- | --- | --- | --- | --- | --- | --- |
|  |  |  |  | Learning Phase | Period 1 | Period 2 |  |
|  | *Recruitment* | *Registration and Baseline* | *Prior to initiate learning phase* | *2 weeks-*  *3 months* | *3 months* | *3 months* |  |
| **Activity/collected data** |  |  |  |  |  |  |  |
| (E)mail invitation to participate in COVID-RED | x |  |  |  |  |  |  |
| Registration and verification of inclusion/exclusion criteria and COVID-19 risk factors |  | x |  |  |  |  |  |
| Informed consent by subject |  | x |  |  |  |  |  |
| Baseline survey (socio-demographic characteristics, comorbidities, medications) |  | x |  |  |  |  |  |
| Assignment of Unique Identification code (UID) and randomization to sequence |  | x |  |  |  |  |  |
| Assignment of login details for Ava COVID-RED app |  | x |  |  |  |  |  |
| Receive starter kit |  |  | x |  |  |  |  |
| Installation of the Ava COVID-RED app, log in and pairing of Ava bracelet |  |  | x |  |  |  |  |
| Collection & testing of plasma/blood sample^1^ |  |  |  | x | x | x | x |
| Daily logging of symptoms and confounders in Ava COVID-RED app |  |  |  |  | | |  |
| Nightly wearing & routine syncing of the Ava bracelet |  |  |  |  | | |  |
| Receive algorithm-based health indicator |  |  |  |  | | |  |
| Testing for presence of SARS-CoV-2 virus^2^ |  |  |  |  | | |  |
| Logging any COVID-19 test results in Ava COVID-RED app^3^ |  |  |  |  | | |  |
| Bi-weekly completion of survey^4^ |  |  |  |  | | |  |
| Interview with subject for HRU or ADE reporting^5^ |  |  |  |  | | |  |
| Return of Ava bracelet |  |  |  |  |  |  | x |

1. ^Fingerprick blood sample is to be taken at start of Learning phase and upon completion of Learning phase, Period 1 and Period 2.^
2. ^Testing is done on the subject’s initiative in line with the governmental rules and additionally based on advice from the AVA COVID-RED app^
3. ^If applicable^
4. ^Survey to obtain more specific details on the testing procedures, COVID-19 vaccination, epidemiological, household, occupational data, behaviour of the subjects and collection of possible hospital episodes related to COVID-19 and adverse device effects (ADEs)^
5. ^If applicable. HRU = Health Resource Utilization, ADE = Adverse Device Effect^

# APPENDIX 2

| **Data Storage** | **Companies with access** | **Does database contain Personal Identifiable information?** | **Study roles with access to this data source** | **Security** |
| --- | --- | --- | --- | --- |
| Castor (EDC, e-Consent, registration) | Julius Clinical, UMCU | Yes, information supplied by participants will be stored here (e.g.: Name, Birth month+year, address, phone number, email) as well as potential ADE and hospitalization event details. | Data manager of JC, Physicians UMCU, Data-entry personnel JC | ISO 27001 (NEN 7510) Certified |
| AVA-servers | AVA | Yes, onboarding app questions contain email address and birthdate. Data from Bracelets, symptoms diary, and reported ADE(s). Only pseudonymized data will be exported. | AVA-employees | GDPR-Compliant, AWS Servers |
| Sanquin/Laboratory database | Sanquin | No, Pseudonymized data only of the COVID-19 test results (serology and PCR) | Lab personnel of Sanquin |  |
| Participant Registry JC | Julius Clinical | Yes, Personal data needed to identify and send study materials from Castor will be stored here. Only Personal data critical to send study-material (i.e. Name, Address) will be shared with Mailstreet | Helpdesk of JC, JC Data Transfer Lead and his/her delegate | Firewalls and virus detection/prevention software and access restriction via authorised username and password restrictions |
| JC Storage* | Julius Clinical | No, Pseudonymized data from Surveys, Hospitalization events. | Authorized COVID-RED employees of JC | Firewalls and virus detection/prevention software and access restriction via authorised username and password restrictions |
|  |  | Yes, all personal identifiable information | Data manager of JC, Helpdesk of JC (excluding test results)  JC Data Transfer Lead and his/her delegate (including test results) |  |
| Mailstreet | Mailstreet | Yes, information critical to send out study-materials (i.e. Name, Address) | Mailstreet employees involved in COVID-RED study | ISO 27001 (information security) certified |
| anDREa Analytics platform | COVID-RED Consortium: UMCU AVA Julius Clinical Vive UCL Takeda | No, Pseudonymized data from AVA (bracelet and symptom diary), Surveys, COVID-19 test results, Hospitalization events | Data scientists/researchers in the COVID-RED study team | GDPR-compliant, Microsoft Azure AD accounts, audit-trails, pen-tested |
| Julius Clinical AWS Analytics platform | COVID-RED Consortium: UMCU AVA Julius Clinical Vive UCL Takeda | No, Pseudonymized data from AVA (bracelet and symptom diary), Surveys, COVID-19 test results, Hospitalization events | Data scientists/researchers in the COVID-RED study team | AWS Servers, Restricted (account/password protected) access, MFA to platform, (audit) log trails, encrypted |

*JC storage will be separated into a storage location with only pseudonymized data and a storage location for Personal identifiable information. Access to the latter will only be provided depending on the study role specified in the fourth column.
